# Development of an Immune-Related Risk Signature for Predicting Prognosis in Lung Squamous Cell Carcinoma

**DOI:** 10.3389/fgene.2020.00978

**Published:** 2020-08-28

**Authors:** Denggang Fu, Biyu Zhang, Lei Yang, Shaoxin Huang, Wang Xin

**Affiliations:** ^1^School of Basic Medicine, Jiujiang University, Jiujiang, China; ^2^School of Medicine, Indiana University, Indianapolis, IN, United States; ^3^School of Pharmacy and Life Science, Jiujiang University, Jiujiang, China

**Keywords:** lung squamous cell carcinoma, immune-related genes, prognosis, signature, risk score

## Abstract

Lung squamous cell carcinoma (LSCC) is the most common subtype of non-small cell lung cancer. Immunotherapy has become an effective treatment in recent years, while patients showed different responses to the current treatment. It is vital to identify the potential immunogenomic signatures to predict patient’ prognosis. The expression profiles of LSCC patients with the clinical information were downloaded from TCGA database. Differentially expressed immune-related genes (IRGs) were extracted using edgeR algorithm, and functional enrichment analysis showed that these IRGs were primarily enriched in inflammatory- and immune-related processes. “Cytokine-cytokine receptor interaction” and “PI3K-AKT signaling pathway” were the most enriched KEGG pathways. 27 differentially expressed IRGs were significantly correlated with the overall survival (OS) of patients using univariate Cox regression analysis. A prognostic risk signature that comprises seven IRGs (GCCR, FGF8, CLEC4M, PTH, SLC10A2, NPPC, and FGF4) was developed with effective predictive performance by multivariable Cox stepwise regression analysis. Most importantly, the signature could be an independent prognostic predictor after adjusting for clinicopathological parameters, and also validated in two independent LSCC cohorts (GSE4573 and GSE17710). Potential molecular mechanisms and tumor immune landscape of these IRGs were investigated through computational biology. Analysis of tumor infiltrating lymphocytes and immune checkpoint molecules revealed distinct immune landscape in high- and low-risk group. The study was the first time to construct IRG-based immune signature in the recognition of disease progression and prognosis of LSCC patients.

## Introduction

Lung cancer is the second most prevalent human malignancy that arises from epithelial cells in both men and women, and is by far the leading cause of cancer death worldwide, accounting for 25% of all cancer deaths. It’s estimated that there are about 228,820 new diagnosed cases and 135,720 deaths from this disease in the United States in 2020 (Siegel et al., 2020). Lung cancers are classified into two main types, non-small cell lung carcinoma (NSCLC) and small cell lung carcinoma (SCLC). Lung squamous cell carcinoma (LSCC) is the second most common histologic type of NSCLC followed adenocarcinoma (LADC) (Fernandez-Cuesta and Foll, 2019), causing about 30% of lung cancers. LSCC is strongly correlated with tobacco smoking than other forms of NSCLC (Cipriano et al., 2019). Although the incidence and mortality continues to decrease partly due to less smoking and advances in early diagnosis and treatment, patients still have a poor prognosis and 5-year survival remains at a very low level (Lu et al., 2019; Thomas et al., 2015). The main treatments of lung cancer are surgical resection and chemotherapy, but vary for various factors, including tumor stage, lung function and genomic alterations. Early stage LSCC patients are typically receiving resected surgically and chemotherapy and/or radiation could be as an adjuvant therapy, while advanced LSCC are given the first-line systematic therapy, commonly a platinum-based regimens (Gandara et al., 2015). Compared to lung adenocarcinoma, much less frequent mutations in EGFR and ALK rearrangements for LSCC postponed the development of targeted therapies (Derman et al., 2015; Yang et al., 2019). Therefore, identification of novel and effective biomarkers will contribute to monitor the progression of LSCC and reduce the numbers of patients that not diagnosed before the onset of this aggressive disease. Many online tools have been developed for identification of biomarkers to assist the diangosis of cancer subtypes and provide survival prediction for cancers based on massive genomic data in recent years (Kamps et al., 2017). As an aggressive disease with leading mortality and incidence worldwide, several databases were constructed to find prognosis related biomarkers in lung cancer, while few online survival analysis softwares available for specific subtypes of lung cancer except than Kaplan-Meier plotter (Gyõrffy et al., 2013) and OSluca (Yan et al., 2020).

Cancer immunotherapy has become a promising treatment for different types of human cancers in recent years. Some immunotherapies have been utilized to leverage the immune system to fight tumors (Kobold et al., 2018; Popovic et al., 2018). Immune checkpoint inhibitors (ICIs), such as anti-PD-1/PD-L1, have emerged as an effective therapeutic selection for advanced breast cancer, metastatic melanoma and NSCLC (Wang et al., 2018; Ahern et al., 2019; Liu D. et al., 2019). NSCLC is characterized by several mutations in the immune system, making it possible that these patients may benefit from immunotherapy. Several monoclonal antibodies targeting the immune checkpoint pathways have been approved for treatment of NSCLC. Anti-PD-1 agent (Nivolumb) could improve the clinical outcome for LSCC. Despite the success of immunotherapy, different patients showed diverse responses, and only a small number of patients benefited from treatment with ICIs agents. The prognostic significance of PD-1 expression in patients with tumors are still controversial. This suggests that it is essential to identify additional predictive biomarkers for developing potential immunotherapy. Different studies proposed models regarding the prognostic utility of immune-related genes in various cancers, including papillary thyroid cancer (Lin et al., 2019), bladder cancer (Song et al., 2019), head and neck squamous cell carcinoma (Cao et al., 2018) and non-squamous NSCLC (Li et al., 2017a). However, the clinical relevance and prognostic significance of IRGs in LSCC has not been well illuminated.

This study aimed to investigate the prognostic utility of IRGs on monitoring the prognosis, and identify novel biomarkers in developing potential targeted therapies for LSCC patients. The gene expression profile and clinical information of LSCC cohort were downloaded from TCGA database. Differentially expressed IRGs were identified, and the prognostic landscape of these IRGs were comprehensively assessed through computational biology. Importantly, we proposed a prognostic signature based on the immunogenomic profile for predicting prognosis in LSCC patients. The study may provide new insights into understanding the functional regulatory mechanisms of IRGs, and help to develop immune-related targeted therapy in the treatment of LSCC in further in-depth work.

## Materials and Methods

### Gene Expression Datasets and IRGs

Level 3 RNA-seq raw count data from 502 lung squamous cell carcinoma patients (LSCC) and 49 non-tumor samples were downloaded from the TCGA database. Clinical information of these patients was also downloaded and extracted. Patients with overall survival (OS) time less than 30 days were removed, and finally 475 patients were employed in further analysis. Immunology Database and Analysis Portal (ImmPort) is a database that updates and shares immunology data accurately and timely (Bhattacharya et al., 2014). More importantly, the database provides a list of IRGs, which were identified to actively participated in the process of immune activity, and were classified into functional categories. A total of 2498 IRGs were derived from the ImmPort for this study (Supplementary Table S1).

### Identification of the Differentially Expressed Genes (DEGs) and IRGs

The differentially expressed genes were identified between the LSCC and adjacent non-tumor samples by *edgeR* R package (Robinson et al., 2010). Genes with | log FC| > 2 and False discovery rate (FDR) < 0.01 were selected as the DEGs. The differentially expressed IRGs were extracted from the DEGs list.

### Heatmap and Clustering Analysis

Heatmap and clustering were performed by the pheatmap R package.

### Gene Functional Enrichment Analysis

Functional enrichment analysis of the DEGs and the differentially expressed IRGs were conducted using clusterProfiler R package (Yu et al., 2012) to identify significantly enriched GO terms, including biological process (BP), molecular function (MF), and cellular components (CC). The pathway analysis with reference from KyotoEncyclopedia of Genes and Genomes pathways (KEGG) was also performed. The *p-*value was adjusted by Benjamini and Hochberg method and less than 0.05 was considered as statistically significant.

### Identification of Overall Survival (OS)-Related IRGs

A log2 (normalized value + 1) expression matrix was used to identify OS-related IRGs by univariate Cox regression analysis using *survival* R package. IRGs that significantly associated with the OS of patients were delivered to further functional analysis and construct the prognostic risk signature.

### Molecular Characteristics of the Differentially Expressed IRGs

To explore the interplay between these OS-related IRGs, the protein-protein interaction (PPI) network was assessed from the STRING database (Franceschini et al., 2013). The PPI network was reconstructed by the Cytoscape software (Shannon et al., 2003).

In addition, transcription factors (TFs) have been known to directly mediate the expression levels of the respective genes. Cistrome Cancer database is an online resource that integrates cancer genomics data from TCGA with over 23,000 profiles of ChIP-seq and chromatin to provide the regulatory interactions between TFs and transcriptomes (Mei et al., 2017). To investigate the potential ability of TFs in regulating these clinically relevant IRGs, a total of 318 TFs were downloaded from Cistrome. The correlation between these IRGs with TFs was calculated, and the Person correlation coefficient greater than 0.3 was set as the cutoff value to construct the regulatory network of the IRGs and potential TFs.

The database of Transcriptional Regulatory Relationships Unraveled by Sentence-based Text mining (TRRUST) was used to identify the key regulated factors of OS-related IRGs. TRRUST is a reliable curated portal for human, and mouse transcriptional regulatory networks, which contains 8,444 TFs-target regulatory relationships of 800 human TFs (Han et al., 2018).

### Construction and Validation of the Immune Related Prognostic Risk Signature for LSCC

OS-related IRGs were employed to construct the prognostic risk signature using multivariate Cox stepwise regression analysis. The minimum number of genes that comprised of the final signature was determined by the Akaike information criterion (AIC) criterion (Vrieze, 2012). The model discrimination performance was assessed by the receiver operating characteristic (ROC) curve using *survivalROC* R package. The patient’s prognostic risk score was calculated based on the corresponding gene expression data multiplied by the Cox regression coefficient. Patients were divided into high- and low-risk group according to the median risk score. The predictive utility of the prognostic signature was evaluated by Kaplan-Meier curve and log rank test.

Subset analysis was conducted to see the utility of the risk score for OS prediction of patients in different clinical parameters set, including age, gender, tumor stage, TNM stage.

To validate the prognostic capability of the immune related risk signature, two independent LSCC cohorts with clinical information, including GSE4573 (*n* = 130) and GSE17710 (*n* = 56), were downloaded from the GEO database. The risk scores and clinical pathological characteristics for LSCC patients from TCGA and these two validation cohorts were summarized in Supplementary Table S2.

### Association of Risk Score With Tumor Immune Landscape Using CIBERSORT and TIMER Database

The CIBERSORT was developed to accurately quantify the abundance of distinct cell types within a complex mixture of gene expression data using deconvolution algorithm (Newman et al., 2015). We performed CIBERSORT analysis to calculate the proportions of 22 immune cell subtypes, including seven T cell types, naïve and memory B cells, plasma cells and NK cells, of LSCC patients in high- and low-risk groups, the samples with *p*< 0.05 were selected for further analysis.

Tumor Immune Estimation Resource (TIMER) is an online database to estimate the abundances of 6 subtypes of tumor infiltrating immune cells in 32 cancer types from TCGA database, including B cells, CD4 T cells, CD8 T cells, macrophages, neutrophils, and dendritic cells via gene expression data. Immune infiltration levels of LSCC patients were calculated, and the Pearson correlation between the risk score and immune cell infiltration was analyzed. Additionally, the correlation of seven-model genes expression with the immune cell infiltration levels in LSCC patients was assessed (GraphPad Prism 8.3.0).

## Results

### Identification of the Differentially Expressed IRGs

**FIGURE 1:**
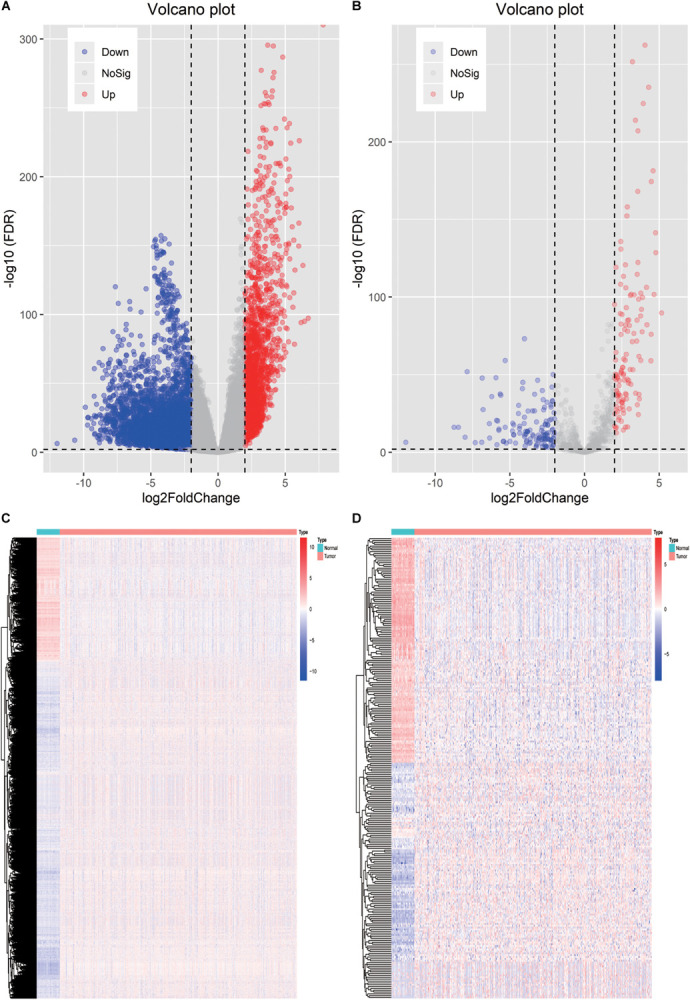
Identification of the differentially expressed immune-related genes. **(A)** Heatmap of differentially expressed genes between LSCC and non-tumors tissues. **(B)** Heatmap of differentially expressed IRGs between LSCC and non-tumors tissues. **(C)** Volcano plot of all the differentially expressed genes. **(D)** Volcano plot of the differentially expressed IRGs.

**FIGURE 2:**
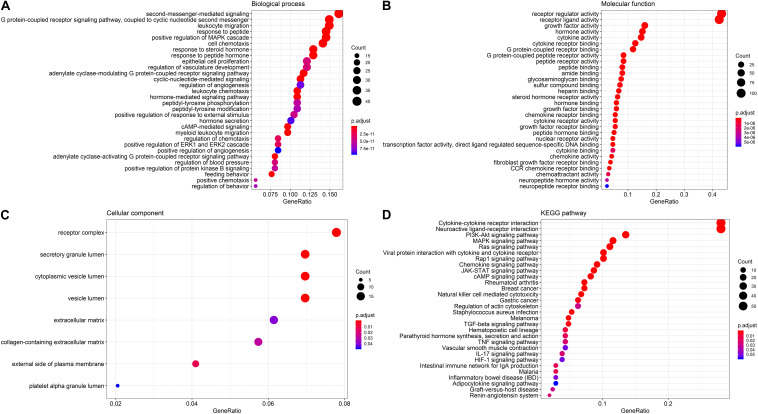
GO terms and pathways analysis of the differentially expressed IRGs. **(A)** The significant enriched biological processes. **(B)** The significant enriched molecular functions. **(C)** The significant enriched cellular components. **(D)** The significant enriched KEGG pathways.

To delineate gene expression profiles between normal and lung squamous cell carcinoma, 6,678 DEGs were identified using the edgeR algorithm (Robinson et al., 2010). Among these DEGs, 4,905 genes were up-regulated and 1,703 genes were down-regulated (Figure 1A). A distinct gene expression pattern was observed in the normal and tumor cases (Figure 1C). 250 IRGs were referenced from ImmPort, including 128 down-regulated and 122 up-regulated (Figure 1B). A similar gene expression difference that defines by IRGs was also apparent in normal and tumor groups (Figure 1D). GO terms analysis showed that these DEGs were significantly enriched in epidermal cell differentiation, keratinization, extracellular matrix and cell-cell injections (Supplementary Figures S1A–C). Cytokine-cytokine receptor interaction and alcoholism ranked the top pathways (Supplementary Figure S1D). As to the IRGs, inflammatory processes were the most frequently implicated through functional enrichment analysis, such as “leukocyte migration,” “cell chemotaxis,” “receptor ligand activity,” “cytokine activity,” and “cytoplasmic vesicle lumen” (Figures 2A–C). “Cytokine-cytokine receptor interaction,” “PI3K-AKT signaling pathway,” and “MAPK signaling pathway” were most enriched pathways by the differentially expressed IRGs (Figure 2D).

### Correlation of Individual Differentially Expressed IRGs With OS

**FIGURE 3:**
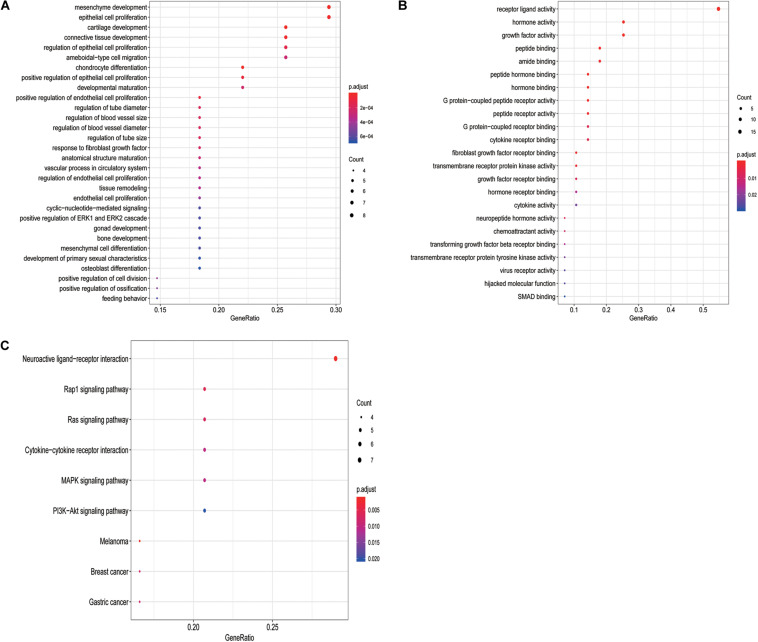
GO terms and pathways analysis of the differentially expressed OS-related IRGs. **(A)** The significant enriched biological processes. **(B)** The significant enriched molecular functions. **(C)** The significant enriched KEGG pathways.

To determine the potential prognostic utility of individual differentially expressed IRGs for patients, the clinical information of LSCC patients was downloaded. 27 differentially expressed IRGs were found to be significantly associated with the OS of LSCC patients (*P* < 0.05, Table 1) using univariate Cox regression analysis. As expected, we found that these OS-associated IRGs were significantly involved in the similar GO terms and pathways that seen in the enrichment analysis using the differentially expressed IRGs, such as “epithelial cell proliferation,” “mesenchyme development,” and “regulation of ERK1 and ERK2 cascade” (Figure 3A). “Hormone activity,” “growth factor activity,” “cytokine receptor binding” (Figure 3B) were the most significant molecular functions. The “Rap1, Ras signaling pathway” and “MAPK signaling pathway” were the top enriched pathways (Figure 3C).

**TABLE 1:** OS-related differentially expressed IRGs in LSCC patients by univariate Cox regression hazard analysis (*P* < 0.05).

Gene	HR	Z	*P*-value
RETN	1.140202	3.264169	0.001098
BMP2	1.161624	3.049499	0.002292
GCGR	0.911235	–2.75875	0.005802
PTH1R	1.15991	2.699299	0.006949
FGF8	0.88845	–2.65865	0.007845
SEMA3B	1.131694	2.61468	0.008931
CLEC4M	1.139322	2.583223	0.009788
PTH	1.177088	2.497194	0.012518
FLT4	1.205449	2.486447	0.012903
SLC10A2	1.138967	2.484331	0.01298
AGRP	1.141067	2.472845	0.013404
NPPC	0.935714	–2.45588	0.014054
PPBP	1.079465	2.412894	0.015826
FGF4	1.089114	2.260932	0.023763
ACVRL1	1.202202	2.24414	0.024823
HGF	1.10855	2.173913	0.029712
NTS	0.966758	–2.1379	0.032525
APLN	1.129638	2.089128	0.036696
GALR3	0.879853	–2.07556	0.037934
FGF9	1.085816	2.070424	0.038413
AMH	0.934864	–2.03968	0.041382
TIE1	1.158155	2.022781	0.043096
EDNRB	1.115529	2.019389	0.043447
TNFSF12	1.184515	2.018298	0.04356
NR0B2	1.075512	1.986496	0.046978
ANGPTL1	1.099632	1.984636	0.047185
ICAM-1	1.110156	1.975964	0.048159

**FIGURE 4:**
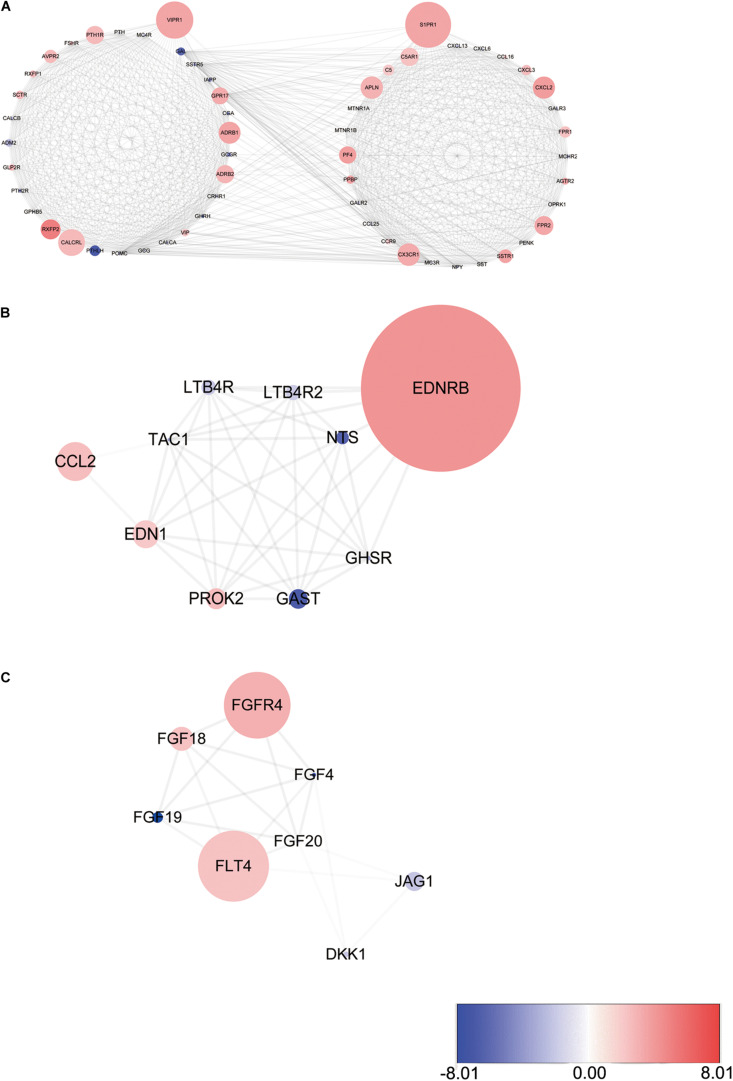
Three modules identified through protein-protein interaction network analysis. **(A)** S1PR1 module. **(B)** EDNRB module. **(C)** FGFR4 module. The color of a node in each module reflects its log transformed fold change, and the circle size represented as adjusted *P*-value.

**FIGURE 5:**
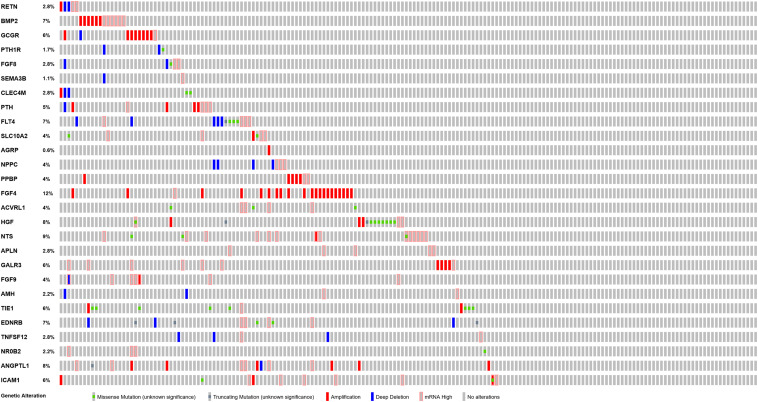
Mutation landscape of OS-related differentially expressed IRGs.

PPI network analysis of these IRGs identified three modules that defined by the number of nodes greater than 10, and named as SIPR1, EDNRB, and FGFR4, respectively. These three module genes were most significantly increased expression in LSCC cases (Figure 4), and have been implicated in cancer cell proliferation and migration (Tanaka et al., 2014; Liu Y. et al., 2019; Huang et al., 2015). In addition, a comprehensive exploration of the molecular characteristics of these OS-related IRGs found that amplification, deep deletion, and mRNA high expression were the three most commonly types of mutations (Figure 5). FGF4 was the gene with the highest mutation frequency, and there were 12 genes with a mutation rate greater or equal to 5%.

### Construction of Transcription Factors (TFs) Regulatory Network

**FIGURE 6:**
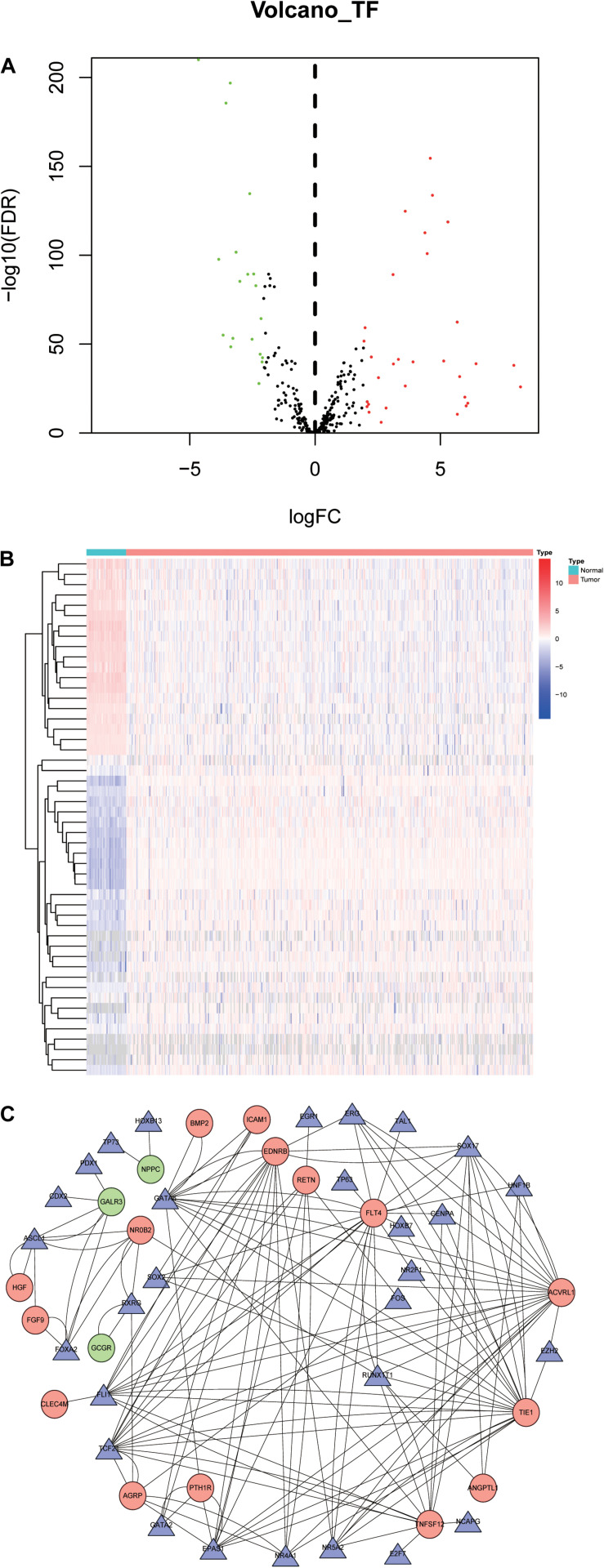
Transcription factors-mediated regulatory network. **(A)** Differentially expressed TFs. **(B)** Heatmap of differentially expressed TFs between LSCC and non-tumors tissues. **(C)** The transcription regulatory network according to the clinically relevant IRGs and differentially expressed TFs. The circle in a node reflects clinically relevant IRGs and triangle represented as differentially expressed TFs. The shades of color reflect the correlation.

To explore the potential molecular mechanisms corresponding to the clinical significance of the OS-related IRGs, the regulatory network of these genes with TFs was investigated. We examined the expression profile of 318 TFs, and found 50 TFs were differentially expressed between LSCC and normal samples (Figures 6A,B). Among these 50 TFs, 3 genes (TCF21, HNF1B, and SOX2) were significantly correlated with the OS of LSCC patients (Supplementary Table S3). A regulatory network based on the Pearson correlation between 27 OS-related IRGs and 50 differentially expressed TFs was constructed using Cytoscape software. A correlation score more than 0.3 was set as the cut-off value. The TFs based regulatory network illustrated the regulatory relationships among these IRGs (Figure 6C).

We identified the key regulated factors of OS-related IRGs using the TRRUST database. Seven key transcription factors (RELA, NFKB1, SP1, VDR, FOS, PPARG and STAT3) were found to be associated with the regulation of these IRGs (Table 2).

**TABLE 2:** Key regulated factor of OS-related IRGs in LSCC patients.

Key TFs	Description	Regulated genes	*P*-value	FDR
RELA	v-rel reticuloendotheliosis viral oncogene homolog A (avian)	FLT4, ICAM1, BMP2, FGF8, AMH	6.04E-05	0.000218
NFKB1	Nuclear factor of kappa light polypeptide gene enhancer in B-cells 1	FLT4, AMH, ICAM1, BMP2, FGF8	6.23E-05	0.000218
SP1	Sp1 transcription factor	ACVRL1, ICAM1, EDNRB, HGF, RETN	0.00049	0.00114
VDR	Vitamin D (1,25- dihydroxyvitamin D3) receptor	AMH, PTH	0.00164	0.00287
FOS	FBJ murine osteosarcoma viral oncogene homolog	SLC10A2, NTS	0.003	0.0042
PPARG	Peroxisome proliferator-activated receptor gamma	RETN, ICAM1	0.004	0.00466
STAT3	Signal transducer and activator of transcription 3 (acute-phase response factor)	HGF, ICAM1	0.0175	0.0175

### Development and Validation of the Immune Related Prognostic Signature

**FIGURE 7:**
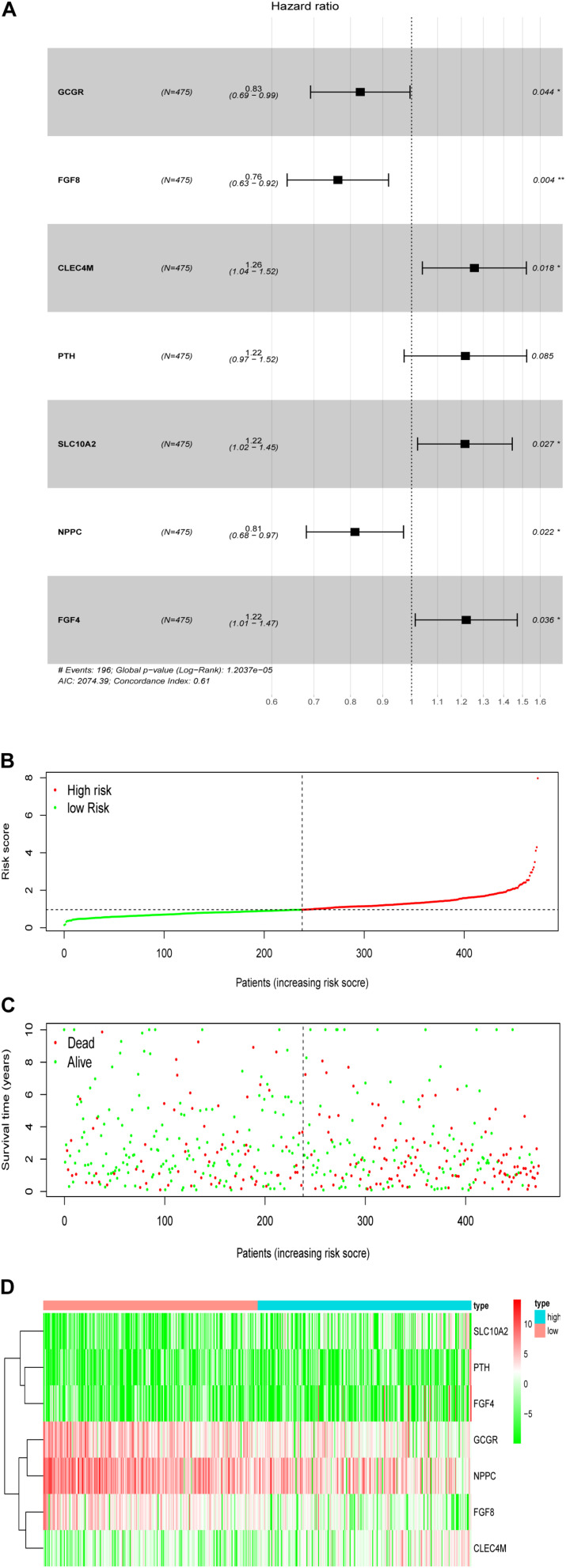
Development of the prognostic signature based on the clinically relevant IRGs. **(A)** The hazard ratio of model genes. **(B)** Distribution of patients’ risk scores. **(C)** Patients’ survival time along with risk score. **(D)** The expression of the seven model genes in high- and low-risk groups.

To establish an optimal prognostic immune related signature to define the patients’ risk, multivariate Cox stepwise regression analysis was performed that employed the 27 OS-related IRGs, we develop a prognostic risk signature that consists of 7 genes (GCCR, FGF8, CLEC4M, PTH, SLC10A2, NPPC, and FGF4) for predicting the OS of patients using the survival R package (Figure 7A). The signature was as follows:

**Prognostic risk score** = [Expression level of *GCCR* *(−0.0855)] + [Expression level of *FGF8* *(−0.1171)] + [Expression level of *CLEC4M* *(0.1312)] + [Expression level of *PTH* *(0.1251)] + [Expression level of *SLC10A2* *(0.1082)] + [Expression level of *NPPC* *(−0.0525)] + [Expression level of *FGF4* *(0.0912)].

**FIGURE 8:**
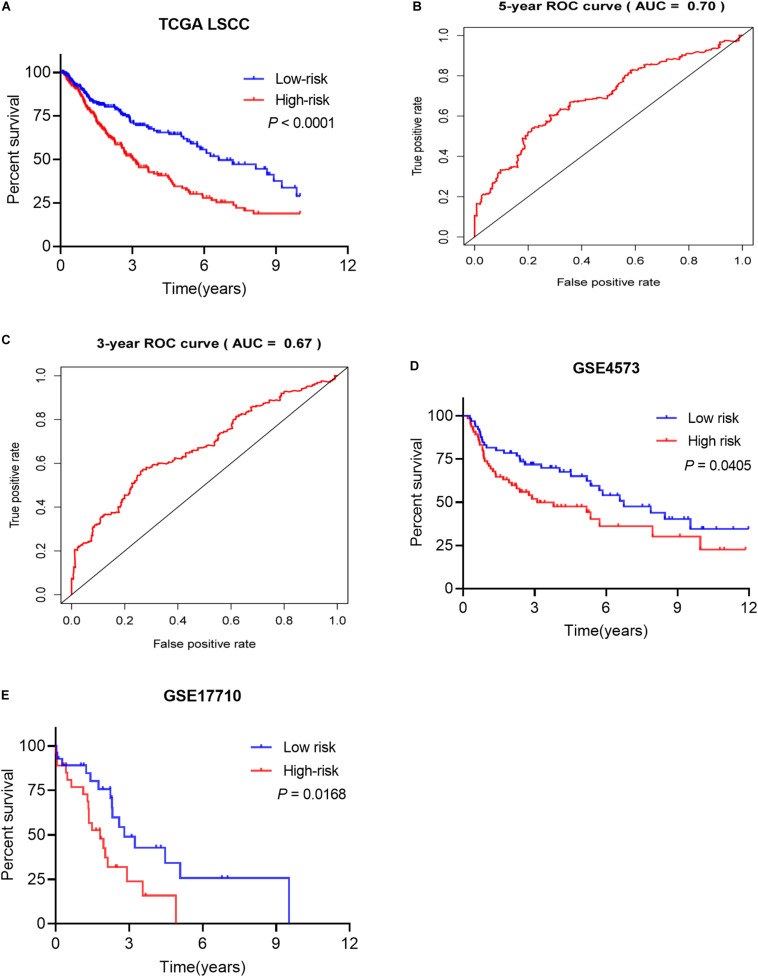
The prognostic signature predicted the OS of LSCC patients. **(A)** Patients in high-risk groups have shorter OS. **(B)** The receiver operating characteristics (ROC) curve of prognostic utility of the signature for 5 years. **(C)** The receiver operating characteristics (ROC) curve of prognostic utility of the signature for 3 years. **(D)** The prognostic utility of the signature in test LSCC cohort (GSE4573, *n* = 130). **(E)** The prognostic utility of the signature in test LSCC cohort (GSE17710, *n* = 56).

Based on the patients’ risk score calculated by the signature, the patients were divided into high- and low-risk groups according to the median value of risk score (Figures 7B,C), and three model genes (GCGR, FGF8 and NPPC) were significantly up-regulated in low-risk patients (Figure 7D and Supplementary Figure S2), while two genes (SLC10A2 and CLEC4M) were markedly down-regulated (Supplementary Figures S2C,E). Additionally, five model genes were up-regulated in LSCC patients (Supplementary Figure S3), while the remaining two genes (SLC10A2 and CLEC4M) were observed to be increased expression in normal cases (Supplementary Figure S3C,E). Kaplan-Meier curve showed that patients in high-risk group have worse OS than that of patients in low-risk group (*P* < 0.0001, Figure 8A). The area under curve (AUC) value of the receiver operating characteristics (ROC) curve was 0.7 (Figure 8B) for 5 years, and 3-year AUC was 0.67 (Figure 8C), suggesting the prognostic signature based on IRGs has moderate capacity for monitoring prognosis. Furthermore, in order to minimize potential over-fitting, we used the least absolute shrinkage and selection operator (Lasso) regression model to select the model genes from 27 IRGs. A signature that comprises of seven IRGs (BMP2, GCGR, PTH, SLC10A2, PPBP, FGF9, and AMH) was constructed using multivariate Cox stepwise regression analysis. The patients were divided into low- and high-risk groups according to the median risk score. The patients in high-risk group have significant shorter OS than that of patients in low-risk group (*P* = 4.2e-03), while the AUC of ROC curve of prognostic utility of this risk signature for 3 and 5 years were 0.62 and 0.61, respectively. This indicated that our signature show better predictive performance for LSCC patients. In addition, we made an attempt to develop several immune related signatures by increasing or decreasing the number of OS-related IRGs. The predictive performance of our signature is superior to these signatures (data not shown).

To evaluate predictive capability of the signature for patients’ prognosis independently, univariate Cox regression analysis found that age, pathological M and T stages, Tobacco smoking history, tumor stage, and patient’s risk score were significantly associated with the OS of LSCC patients (Table 3). Multivariate Cox regression analysis showed that the risk signature could serve as an independent predictor after adjusting for other clinical parameters, including age, gender, tumor stage, pathological stage, tobacco smoking history and cigarette exposure per day (Table 3).

**TABLE 3:** Univariate and multivariate Cox regression analysis of the risk score and clinical parameters in LSCC patients.

Variables	Univariate analysis		Multivariate analysis	
	HR (95% CI)	*P*-value	HR (95% CI)	*P*-value
Age	1.0189 (1.0042–1.0338)	0.0116	1.0158 (1.0007–1.0311)	0.0405
pM	1.2810 (1.0533–1.5580)	0.0131	1.2609 (1.0328–1.5395)	0.0228
pN	1.1680 (0.9839–1.3866)	0.0761	1.0929 (0.8826–1.3533)	0.415
pT	1.3538 (1.1272–1.6259)	0.0012	1.3019 (1.0287–1.6476)	0.0281
Gender	0.8031 (0.5752–1.1213)	0.1978	0.8395 (0.5981–1.1783)	0.3118
Tobacco_smoking_history	0.8829 (0.7805–0.9987)	0.0476	0.8667 (0.7634–0.9841)	0.0273
Tumor_stage	1.2859 (1.1008–1.5020)	0.0015	1.0861 (0.8593–1.3729)	0.4895
Tigarettes_exposures (per_day)	1.0021 (0.9295–1.0803)	0.9571	1.0079 (0.9345–1.0870)	0.8388
Risk score	1.5196 (1.3462–1.7155)	1.32E–11	1.5480 (1.3577–1.7649)	6.62E-11

The proposed signature was tested using external LSCC cohorts from GSE4573 (*n* = 130) and GSE17710 (*n* = 56). The risk scores of patients were calculated. Patients were divided into high- and low-risk group according to the median risk score in both validation datasets. Patients in high-risk group have significant shorter OS than that of patients in low-risk group in GSE4573 (Figure 8D, *P* = 0.0405). Similar observation was seen in GSE17710 LSCC cohort (Figure 8E, *P* = 0.0168) both validation cohorts. This demonstrated that our signature has good ability of risk stratification for LSCC patients.

In addition, the tumor infiltration levels among the somatic copy number alternations category (deep deletion, arm-level deletion, diploid/normal, arm-level gain, and high amplification) for the model genes varies in each immune subset compared with the normal using two sided Wilcoxon rank sum test (Supplementary Figures S4A–G).

### The Utility of the Prognostic Signature in OS Prediction for Patients With Different Clinicopathological Factors

**FIGURE 9:**
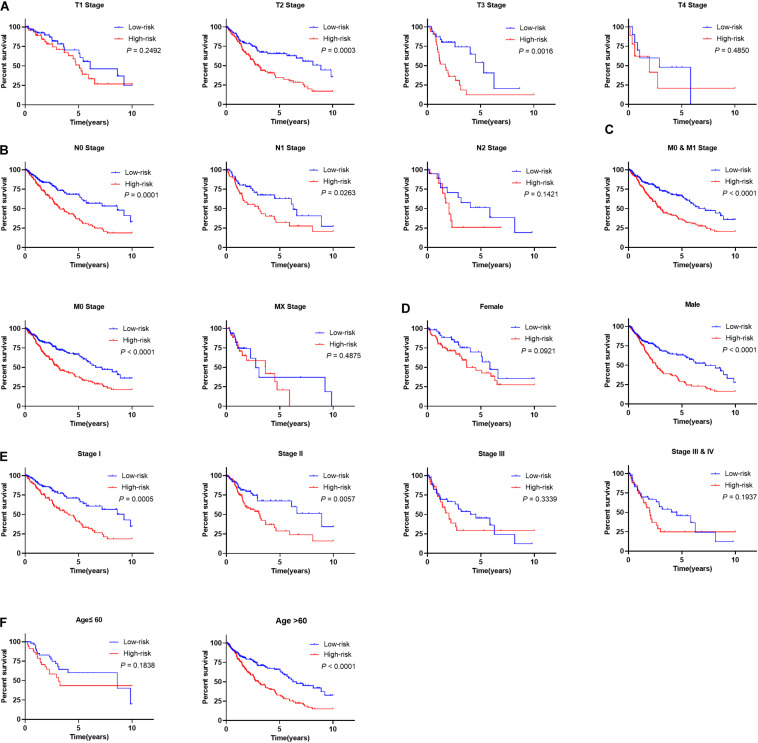
Prognostic utility of signature in LSCC patients with different clinical parameters. **(A)** The prognostic utility of the signature in LSCC patients with different T stages. **(B)** The prognostic utility of the signature in LSCC patients with different node statuses. **(C)** The prognostic utility of the signature in LSCC patients with different M stages. **(D)** The prognostic utility of the signature in male and female LSCC patients. **(E)** The prognostic utility of the signature in LSCC patients with different clinical tumor stages. **(F)** The prognostic utility of the signature in LSCC patients with different age groups.

The subset analysis was performed to determine the utility of our signature in predicting patient’s OS in different clinicopathological parameters. According to the Kaplan-Meier analysis, the risk score has potential prognostic values for the different subsets of LSCC patients (Figures 9A–F), such as patients with pathological T2 and T3 stages, N0-1 stages, M0-1, early tumor clinical stages (stage 1/2), age greater than 60 years old, and male patients. Patients with high-risk patients did have significantly worse outcome than those of low-risk groups. Furthermore, the risk score in high- and low-risk groups with different clinical parameters show significant difference (Supplementary Figures S5A–D).

### The Relevance of the Prognostic Signature and Tumor Immune Landscape

**FIGURE 10:**
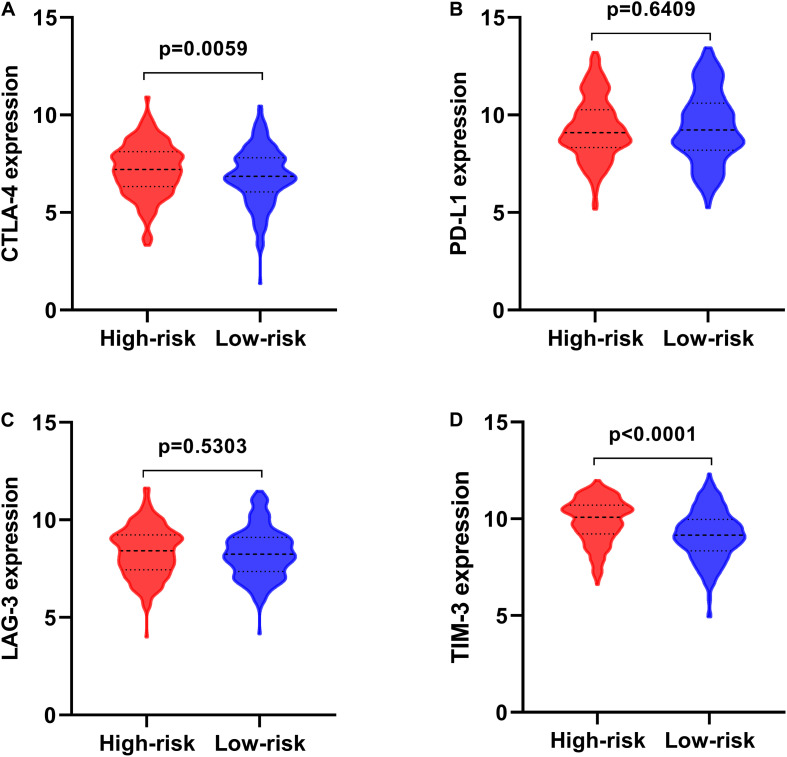
The expression of immune checkpoint molecules in high- and low-risk groups. **(A)** CTLA-4; **(B)** PD-L1; **(C)** LAG-3; **(D)** TIM-3.

Much attention has been paid to develop anti-tumor immune therapies for lung cancers recently, and major advances have been made, especially for immune checkpoint blockade, such as anti-PD1 antibodies Nivolumab and Pembrolizumab (Tanaka et al., 2014; Sul et al., 2016), and anti-PD-L1 Atezolizumab (No Authors Listed, 2016). The expression of immune checkpoint molecules, involving cytotoxic T-lymphocyte-associated protein 4 (CTLA4), programmed cell death 1 ligand (PD-L1), lymphocyte activation gene-3 (LAG-3), and T cell immunoglobulin-3 (TIM-3) of LSCC patients stratified by the prognostic signature, showed that CTLA4 and TIM-3 were significantly increased expression in high-risk patients (Figures 10A–D), suggesting patients in high-risk group might have poor response to the targeted molecular immunotherapy.

**FIGURE 11:**
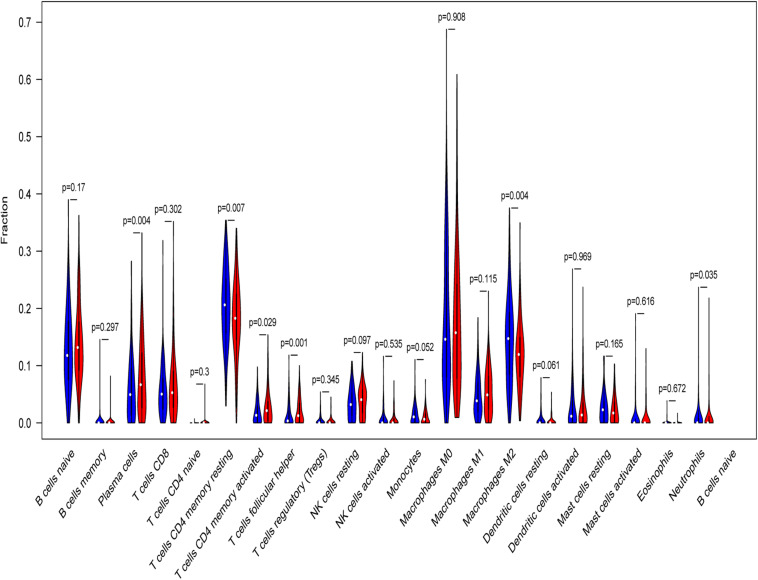
Relative infiltrating proportion of immune cells in high- and low-risk groups. Blue violin reflects high-risk groups, and red violin represents low-risk groups.

Tumor infiltrating lymphocytes (TILs) have been proposed to play a vital role in regulating tumor immune microenvironment (TME), treatment response and clinical outcome. CIBERSORT was applied to calculate the proportions of 22 immune cells types in high- and low-risk LSCC patients. Low-risk patients had remarkably higher fraction of plasma cells, memory activated CD4 T cells, and follicular helper T cells, whereas CD4 memory resting T cells, monocytes cells, macrophages M2 and neutrophils were in high levels (Figure 11).

**FIGURE 12:**
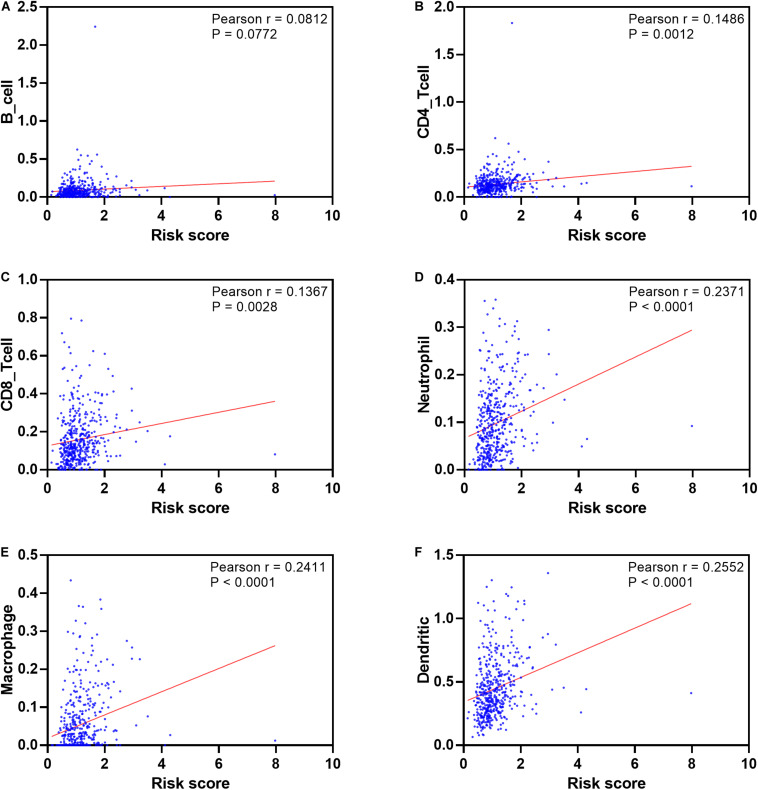
Pearson’s correlation of the risk score and infiltration abundance of six types of immune cells. **(A)** B cell; **(B)** CD4 cell; **(C)** CD8 cell; **(D)** neutrophil cell; **(E)** macrophage cell; **(F)** dendritic cell.

To see if the immunogenome accurately reflected the status of TME, we found that the infiltration level of five immune cell types (Figures 12A–F), including CD4 T cell, CD8 T cell, neutrophil, macrophage and dendritic cells, are significantly associated with patient’ risk score. In addition, infiltration levels of macrophage and dendritic cells ranked the top among the immune cells of the seven model genes (Supplementary Figures S6A–G).

## Discussion

Squamous cell carcinoma is one of the most frequently diagnosed histologic subtype of NSCLC, representing 20–30% of all the NSCLC cases (Travis, 2020). The majority of patients with this disease were closely correlated with the history of cigarette smoking, although the condition is improved alongside cancer incidence. The significance of TME in cancer development, progression and treatment response has been attracted great attention (Chen and Mellman, 2017). Patients with LSCC have benefited from an expanding immunotherapies, such as programmed cell death-1 (PD-1)/programmed cell death ligand 1 (PD-L1) and cytotoxic T-lymphocyte-associated protein 4 (CTLA-4) inhibition, VEGFR inhibition, and targeted therapies matched to fibroblast growth factor receptor (FGFR) and PI3K-AKT (Derman et al., 2015). However, only a small number of patients showed effective remission to immune checkpoint blockade (Wei et al., 2018) or chimeric antigen receptor (CAR) T cell therapy (Mohanty et al., 2019). Identification of strong gene signature to trace the immune status of cancer patients would be vital to establish reliable immune prognostic biomarkers, and to enable stratification of patients into high- and low-risk groups that might be beneficial or responsive to immunotherapy. Several gene signatures that could be representative of tumor immune status have been proposed, and demonstrated to have potential prognostic utility in some cancers, such as non-squamous NSCLC (Li et al., 2017a), hepatocellular carcinoma (Long et al., 2019), renal papillary cell carcinoma (Wang et al., 2019), and renal cancer (Geissler et al., 2015). Although insights into the roles of IRGs in tumor progression and immunotherapeutic have been seen in many types of cancers, a comprehensive and transcriptome-wide profile investigation for their clinical significance and molecular mechanisms in LSCC are yet to be described. In the present study, we developed a prognostic risk signature that comprised of seven IRGs, which demonstrated to be a reliable predictor in identifying LSCC patients with an unfavorable prognosis. In addition, the clinical significance and potential tumor immune landscapes of these IRGs in LSCC patients were also well illuminated.

GO terms enrichment analysis indicated that these differentially expressed IRGs are mainly involved in the process of inflammatory responses, such as “leukocyte migration,” “cell chemotaxis,” “cytokine activity.” “PI3K-AKT signaling pathway” and “cytokine-cytokine receptor interaction” were significantly enriched in the LSCC progression, which is line with previous studies reported that these biological processes and pathways play a crucial role in the proliferation, angiogenesis, immune responses and progression in various cancers (Pons-Tostivint et al., 2017; Zhang et al., 2017; Gao J. et al., 2018). Cancer progression is associated with a pro-inflammatory environment (Lin and Karin, 2007; Lippitz, 2013). Dysregulation of cytokine interactions was involved in the pathogenesis of lung cancer (Zhang et al., 2016; Ozawa et al., 2019). High cytoplasmic RAP1 can increase cisplatin resistance of NSCLC combined with increased NF-κB activity (Xiao et al., 2017), and RAS-RAF-MEK-ERK signaling was a vital pathway that mediates ALK positive tumor cell survival in lung cancer (Hrustanovic and Bivona, 2016). ALK inhibitors, such as crizotinib and ceritinib, have been applied to treat the ALK positive subset of patients (Hrustanovic et al., 2015). These differentially expressed IRGs in our study provided the clues of densely infiltrated inflammatory microenvironment that occurs often in the initiation of cancer cells, and the correlations between activation of immune-related pathways and disease progression and treatment response. Univariate Cox regression analysis showed that 27 IRGs were significantly correlated with patient’s OS, suggesting that these genes could be prognostic biomarkers for their potential predictive utility. Further exploration of these OS-related IRGs observed that amplification, deep deletion, and mRNA high expression were the dominantly molecular traits. For example, Bone morphogenetic protein-2 (BMP2) is overexpressed in the majority of human lung carcinomas independent of cell types (Langenfeld et al., 2005). Previous study indicated that the expression pattern induced by BMP2 in lung fibroblasts may predicts patients’ prognosis in lung adenocarcinoma (Rajski et al., 2015). Consistent with previous evidence, interleukin-33 (IL33) and interleukin 1 receptor-like 1 (IL1RL1) were found to be increased in lung cancer and associated with disease clinical stage (Wang et al., 2016). IL-33/ST2 signaling pathway has been implicated in tumor-associated immune response and inflammatory disease of the lung (Casciaro et al., 2019), and IL-33 could significantly promote the migration and invasion of lung cancer cells through alpha serine/threonine-protein kinase (AKT) pathway activation (Yang et al., 2018). As an attractant, IL-33 can promote the recruitment of Th2-associated cytokines, which act as key mediators for the recruitment of neutrophils, monocytes, NK cells, dendritic cells or T lymphocytes in inflammatory conditions at the site of tumor (Brabcova et al., 2014). Some chemokines were increased in LSCC samples, such as CCL2, CCL15, CCL16, and these chemokines were implicated in the angiogenesis or angiostasis balance and promoted tumor infiltrating hematopoietic cells in the pathophysiology of NSCLC (Rivas-Fuentes et al., 2015). The interaction network among these IRGs and the impact of the mutations might shape the LSCC immune microenvironment. In turn, the TME mediated disease progression and the response to treatment.

To explore the potential molecular mechanisms, a TF-mediated network was used to identify vital TFs that might regulate these IRGs. S1PR1, EDNRB and FGFR4 were predominantly signatures that represented the modules. They are tightly correlated with cancer development and progression (Tanaka et al., 2014; Lang and Teng, 2019; Liu Y. et al., 2019), and involved in the egress of T cells from lung tissue in tumor infiltrating lymphocytes conditions (Mackay et al., 2015). Seven TFs were found to be key regulators of these OS-related IRGs. Of these TFs, as a subunit of NF-κB complex, the phosphorylation of RELA was associated with disease progression, inflammatory regulation and various cancers through NF-κB signaling pathway (Lu and Yarbrough, 2015). NFKB1 exerted an inhibitory function in the tumorigenesis and progression of different types of cancers through alleviating the abnormal activation of the NF-κB signaling (Concetti and Wilson, 2018). NF-κB activity in lung cancer was significantly associated with T cell infiltration, suggesting this pathway may mediate immune surveillance and promote antitumor T cell response (Hopewell et al., 2013). Our results may provide some clues that these IRGs (FLT4, AMH, ICAM1, BMP2, and FGF8) regulated by NFKB1 could be potentially implicated in the immune regulation of LSCC. Growing evidence has indicated that STAT3 signaling was involved in carcinogenesis, and immunotherapy response through regulating cancer cell differentiation and proliferation in human squamous cell carcinoma (Zhou et al., 2015; Zhao et al., 2018). Growing evidence showed that ICAM-1, a specific ligand for leukocyte-function associated antigen-1, interleukin-1, tumor necrosis factor-1 and interferon-γ, participates in various inflammatory immune processes through stabilizing T cell receptor-mediated binding between antigen-presenting cells and T-lymphocytes, including cell-cell interaction and leukocyte transmigration (Kotteas et al., 2014). ICAM-1 blockade inhibited lung cancer cell invasion *in vitro* and tumor metastasis *in vivo*. Tumor necrosis factor-α (TNF-α) induced ICAM-1 can be suppressed by thalidomide administration through inhibition of NF-κB binding to the ICAM-1 promoter (Lin et al., 2006). This suggested targeting ICAM-1 could be a potentially effective therapy for LSCC patients. Previous studies provided limited direct information about these TF-mediated differentially expressed IRGs in LSCC development. Hence, the immunomodulatory role of these genes in monitoring LSCC progression and prognosis prediction remain to be fully investigated.

Recent years have witnessed a boom of gene signatures in clinical practice. Oncotype DX provided a breast cancer recurrence score based on the expression of 21 genes (Siow et al., 2018), and Colorant also represented a recurrence score tool for colon cancer patients constructed by the expression of 18 genes (Tan and Tan, 2011). These signatures developed based on gene expression profiles suggested that screening new prognostic cancer biomarkers is a promising approach to identify high-risk patients for subsequent clinical decision-making, such as therapy selection, outcome prediction and disease progression tracking. As regard to lung cancer, Wan et al. (2012), identified a smoking-associated 7-gene signature for predicting patient’s diagnosis and prognosis in smokers. Similarly, the authors also developed a smoking-associated 6-gene signature that predicts patient’s risk and survival by modeling crosstalk with major lung cancer signaling pathways (Guo and Wan, 2012). Zhang et al. (2019), constructed a 5-microRNA signature based on serum microRNA profiling to predict survival for patients with advanced-stage NSCLC. However, there are few rigorously classifiers that use to predict prognosis for LSCC patients. Although 14-gene signature was developed for LSCC prognosis, the moderate large number of genes make it difficult for clinical use (Li et al., 2017b). Here, we developed an immune related prognostic risk signature with seven OS-related IRGs for LSCC patients. Association analysis combining with the model risk score and clinicopathological characteristics indicated that a higher risk score was significantly correlated with advanced stage, older age and poor prognosis. This is in keeping with higher risk score representing an immunosuppressive tumor microenvironment. This, in turn, promotes tumor progression and worse response to therapies. The seven-gene signature remained an independent prognostic predictor after adjusting for clinicopathological variables. Considering that a robust prognostic signature could be risk-stratify indicator in other independent cohorts, we used two LSCC datasets to validate the predictive efficacy, and found that the signature performed well in classifying high- and low-risk prognostic groups (*P* = 0.0405 for GSE4573, and *P* = 0.0168 for GSE17710). Additionally, our prognostic signature has a moderate high AUC using seven genes when compared to other signatures developed by greater than or less than seven IRGs, which make it more implementable in clinical practice.

Immune escape of tumor cells is a big bugbear to antitumor immune response during cancer progression (Dunn et al., 2002). This process was mediated by several immunosuppressive mechanisms, including increased immunosuppressive cells, and overexpression of immune checkpoint molecules (e.g., PD-1, CTLA-4, LAG3, and TIM-3) in the TME. In this study, the immune cell infiltration landscape in high- and low-risk LSCC patients and the correlation between risk score with the expression of immune checkpoint genes, including PD-L1, CTLA-4, LAG3, and TIM-3 (Woo et al., 2012), were assessed. Consistent with TIM-3 was up-regulated in LSCC and positively correlated with malignant parameters (Gao J. et al., 2018). Our analysis indicated that CTLA-4 and TIM-3 expression were significantly higher in high-risk LSCC groups. Patients in the low-risk group possessed higher plasma cells, CD4 memory activated T cells, T follicular helper cells, macrophage M0 and M1 as well as other immune cells infiltration. Clinical studies demonstrated that tumor-infiltrating lymphocytes (TILs) have a major impact on the disease progression in various cancers (Al-Shibli et al., 2008; Granetto et al., 2017), and increased infiltration of TILs, such as cytotoxic T cells, memory T cells, and T helper cells 1, was associated with favorable prognosis (Bremnes et al., 2016). Previous reports revealed that different subsets of cells differentiating from CD4 + T-cells can promote the inactivation of CD8 T cells, the killing effect of NK cells, enhance the cytotoxicity of CD8 T cells in immune response (Pinto et al., 2018). Significant positive association of six types of immune cell infiltration with risk score indicated that high-risk LSCC patients tended to have more CD4, CD8, neutrophil, macrophages and dendritic cells than patients in low-risk group. This may mean the anti-tumor response in high T cell infiltration is neutralizing through immunosuppressive TME shaped by increased expression of immune checkpoint molecules. These results might help to explain poor prognosis of high-risk patients, and the seven-gene risk signature may provide the potential immunotherapeutic insight for immune checkpoint inhibitor therapies, while the underlying interactions of the IRGs and immune mediators in LSCC need further identification.

Among the seven genes, the expression level of four genes (GCGR, FGF8, CLEC4M, and SLC10A2) were associated with patients’ OS using the median expression value as cutoff in OSluca database (Supplementary Figure S7). CLEC4M, SLC10A2 and FGF4 have been found to be involved in lung cancer progression and the regulation of treatment resistance. CLEC4M is associated with worse prognosis and inhibition of CLEC4M showed potential clinical relevance in counterbalancing cisplatin resistance in NSCLC patients (Tan et al., 2019). Fibroblast growth factor (FGF) isoforms, such as FGF4, FGF19 and FGF7, promoted epithelial-mesenchymal transition (EMT), cell proliferation and migration during cancer progression by inducing store-operated calcium entry in lung carcinoma (Qi et al., 2016). In addition, combination therapy of tyrosine kinase inhibitors, such as FGFR and CFS1R inhibitors, with anti-PD-1 or anti-CTLA-4 antibodies showed promising benefit for cancer patients (Katoh, 2016). As a protective factor, increased expression of SLC10A2 is closely related to suppression of NSCLC cell proliferation and migration, and promote apoptosis under bexarotene treatment (Ai et al., 2018). Consistent with evidence that reduction of glucagon receptor, GCGR, in papillary thyroid carcinoma resulted in the inactivation of EMT and P38/ERK pathways (Jiang et al., 2020), GCGR was down-regulated in LSCC and may serve as a potential prognostic biomarker and therapeutic target for LSCC. NPPC and PTH have not been previously reported being related to lung cancer, PTH related protein mediated energy wasting in fat tissues, and neutralization of this protein can ameliorate cancer cachexia, which improves patient’s survival (Kir et al., 2014). Our study is the first time to suggest their predictive potential as prognostic markers for LSCC patients.

## Conclusion

In the present study, we developed a seven-gene risk signature for LSCC patients based on differentially expressed IRGs, which could serve as an independent prognostic predictor, and an indicator of tumor immune landscape. The patients divided by the risk score have markedly different prognoses. Correlation analysis of the risk score with clinicopathological factors showed that the signature has potential utility of estimating LSCC patients’ prognosis and guiding clinical use. The data presented may provide insight into developing novel therapeutic strategies. Further work should concentrate on uncovering the molecular mechanisms of these IRGs in regulating LSCC progression and outcome, as well as validating our signature in clinical setting.

## Data Availability Statement

The data analyzed in this study are available in the following repositories: TCGA: https://portal.gdc.cancer.gov/; GEO: https://xenabrowser.net/datapages/; TIMER: https://cistrome.shinyapps.io/timer/; STRING: https://string-db.org/cgi/input.pl/; Cistrome Cancer: http://cistrome.org/CistromeCancer/; TRRUST: https://www.grnpedia.org/trrust/.

## Author Contributions

DF: conceptualization, design, and writing – review and editing. DF, BZ, and SH: data acquisition. DF, BZ, LY, SH, and WX: methodology. DF and BZ: data analysis, interpretation, and writing – original draft. DF and WX: project administration. All authors contributed to the article and approved the submitted version.

## Conflict of Interest

The authors declare that the research was conducted in the absence of any commercial or financial relationships that could be construed as a potential conflict of interest.

## Abbreviations

CIBERSORT: Cell-type Identification By Estimating Relative Subsets Of RNA Transcripts
DEGs: differentially expressed genes
GEO: Gene Expression Omnibus
IRGs: Immune-related genes
LSCC: lung squamous cell carcinoma
NSCLC: non-small cell lung carcinoma
PPI: protein-protein interaction
ROC: the receiver operating characteristics curve
TCGA: The Cancer Genome Atlas
TILs: Tumor infiltrating lymphocytes
TIMER: Tumor Immune Estimation Resource
TME: Tumor microenvironment.

## References

[B1] Ahern E. Cubitt A. Ballard E. Teng M. W. L. Dougall W. C. Smyth M. J. (2019). Pharmacodynamics of Pre-Operative PD1 checkpoint blockade and receptor activator of NFkB ligand (RANKL) inhibition in non-small cell lung cancer (NSCLC): study protocol for a multicentre, open-label, phase 1B/2, translational trial (POPCORN). *Trials* 20:753. 10.1186/s13063-019-3951-x PMC6924018 31856909

[B2] Ai X. Mao F. Shen S. Shentu Y. Wang J. Lu S. (2018). Bexarotene inhibits the viability of non-small cell lung cancer cells via slc10a2/PPARgamma/PTEN/mTOR signaling pathway. *BMC Cancer* 18:407. 10.1186/s12885-018-4224-x 29642873 PMC5896077

[B3] Al-Shibli K. I. Donnem T. Al-Saad S. Persson M. Bremnes R. M. Busund L. T. (2008). Prognostic effect of epithelial and stromal lymphocyte infiltration in non-small cell lung cancer. *Clin. Cancer Res.* 14 5220–5227. 10.1158/1078-0432.ccr-08-0133 18698040

[B4] Bhattacharya S. Andorf S. Gomes L. Dunn P. Schaefer H. Pontius J. (2014). ImmPort: disseminating data to the public for the future of immunology. *Immunol. Res.* 58 234–239. 10.1007/s12026-014-8516-1 24791905

[B5] Brabcova E. Kolesar L. Thorburn E. Striz I. (2014). Chemokines induced in human respiratory epithelial cells by IL-1 family of cytokines. *Folia Biol.* 60 180–186. 10.14712/fb2014060040180 25152051

[B6] Bremnes R. M. Busund L. T. Kilvaer T. L. Andersen S. Richardsen E. Paulsen E. E. (2016). The role of tumor-infiltrating lymphocytes in development, progression, and prognosis of non-small cell lung cancer. *J. Thorac. Oncol.* 11 789–800. 10.1016/j.jtho.2016.01.015 26845192

[B7] Cao B. Wang Q. Zhang H. Zhu G. Lang J. (2018). Two immune-enhanced molecular subtypes differ in inflammation, checkpoint signaling and outcome of advanced head and neck squamous cell carcinoma. *Oncoimmunology* 7:e1392427. 10.1080/2162402x.2017.1392427 29308323 PMC5749623

[B8] Casciaro M. Cardia R. Di Salvo E. Tuccari G. Ieni A. Gangemi S. (2019). Interleukin-33 involvement in nonsmall cell lung carcinomas: an update. *Biomolecules* 9:203. 10.3390/biom9050203 31130612 PMC6572046

[B9] Chen D. S. Mellman I. (2017). Elements of cancer immunity and the cancer-immune set point. *Nature* 541 321–330. 10.1038/nature21349 28102259

[B10] Cipriano E. Tavares A. Soares A. Estevinho F. Sottomayor C. (2019). Non-small cell lung cancer in the elderly: a retrospective study comparing first-line treatment with single-agent vs combination chemotherapy vs tyrosine kinase inhibitor. *Ann. Oncol.* 30(Suppl. 2):ii75 10.1093/annonc/mdz065.005

[B11] Concetti J. Wilson C. L. (2018). NFKB1 and cancer: friend or foe? *Cells* 7:133. 10.3390/cells7090133 30205516 PMC6162711

[B12] Derman B. A. Mileham K. F. Bonomi P. D. Batus M. Fidler M. J. (2015). Treatment of advanced squamous cell carcinoma of the lung: a review. *Transl. Lung Cancer Res.* 4 524–532. 10.3978/j.issn.2218-6751.2015.06.07 26629421 PMC4630512

[B13] Dunn G. P. Bruce A. T. Ikeda H. Old L. J. Schreiber R. D. (2002). Cancer immunoediting: from immunosurveillance to tumor escape. *Nat. Immunol.* 3 991–998. 10.1038/ni1102-991 12407406

[B14] Fernandez-Cuesta L. Foll M. (2019). Molecular studies of lung neuroendocrine neoplasms uncover new concepts and entities. *Transl. Lung Cancer Res.* 8 S430–S434. 10.21037/tlcr.2019.11.08 32038931 PMC6987337

[B15] Franceschini A. Szklarczyk D. Frankild S. Kuhn M. Simonovic M. Roth A. (2013). STRING v9.1: protein-protein interaction networks, with increased coverage and integration. *Nucleic Acids Res.* 41 D808–D815. 23203871 10.1093/nar/gks1094 PMC3531103

[B16] Gandara D. R. Hammerman P. S. Sos M. L. Lara P. N. Jr. Hirsch F. R. (2015). Squamous cell lung cancer: from tumor genomics to cancer therapeutics. *Clin. Cancer Res.* 21 2236–2243. 10.1158/1078-0432.ccr-14-3039 25979930 PMC4862209

[B17] Gao J. Qiu X. Li X. Fan H. Zhang F. Lv T. (2018). Expression profiles and clinical value of plasma exosomal Tim-3 and Galectin-9 in non-small cell lung cancer. *Biochem. Biophys. Res. Commun.* 498 409–415. 10.1016/j.bbrc.2018.02.114 29452091

[B18] Gao Y. Yang J. Cai Y. Fu S. Zhang N. Fu X. (2018). IFN-gamma-mediated inhibition of lung cancer correlates with PD-L1 expression and is regulated by PI3K-AKT signaling. *Int. J. Cancer* 143 931–943. 10.1002/ijc.31357 29516506

[B19] Geissler K. Fornara P. Lautenschlager C. Holzhausen H. J. Seliger B. Riemann D. (2015). Immune signature of tumor infiltrating immune cells in renal cancer. *Oncoimmunology* 4:e985082. 10.4161/2162402x.2014.985082 25949868 PMC4368143

[B20] Granetto C. Ricci V. Fea E. Vivenza D. Lo Nigro C. Fortunato M. (2017). Correlation between the prognostic value of tumor-infiltrating lymphocytes (TILS) and sidedness in colorectal cancer (CC) patients (pts). *Ann. Oncol.* 28(Suppl. 3):iii94 10.1093/annonc/mdx261.267

[B21] Guo N. L. Wan Y. W. (2012). Pathway-based identification of a smoking associated 6-gene signature predictive of lung cancer risk and survival. *Artif. Intell. Med.* 55 97–105. 10.1016/j.artmed.2012.01.001 22326768 PMC3351561

[B22] Gyõrffy B. Surowiak P. Budczies J. Lánczky A. (2013). Online survival analysis software to assess the prognostic value of biomarkers using transcriptomic data in non-small-cell lung cancer. *PLoS One* 8:e82241. 10.1371/journal.pone.0082241 24367507 PMC3867325

[B23] Han H. Cho J. W. Lee S. Yun A. Kim H. Bae D. (2018). TRRUST v2: an expanded reference database of human and mouse transcriptional regulatory interactions. *Nucleic Acids Res.* 46 D380–D386. 10.1093/nar/gkx1013 29087512 PMC5753191

[B24] Hopewell E. L. Zhao W. Fulp W. J. Bronk C. C. Lopez A. S. Massengill M. (2013). Lung tumor NF-kappaB signaling promotes T cell-mediated immune surveillance. *J. Clin. Invest.* 123 2509–2522. 10.1172/jci67250 23635779 PMC3668836

[B25] Hrustanovic G. Bivona T. G. (2016). RAS signaling in ALK fusion lung cancer. *Small GTPases* 7 32–33. 10.1080/21541248.2015.1131803 26901483 PMC4905280

[B26] Hrustanovic G. Olivas V. Pazarentzos E. Tulpule A. Asthana S. Blakely C. M. (2015). RAS-MAPK dependence underlies a rational polytherapy strategy in EML4-ALK-positive lung cancer. *Nat. Med.* 21 1038–1047. 10.1038/nm.3930 26301689 PMC4734742

[B27] Huang H. P. Feng H. Qiao H. B. Ren Z. X. Zhu G. D. (2015). The prognostic significance of fibroblast growth factor receptor 4 in non-small-cell lung cancer. *Onco Targets Ther.* 8 1157–1164. 10.2147/OTT.S81659 26045670 PMC4447177

[B28] Jiang H. C. Chen X. R. Sun H. F. Nie Y. W. (2020). Tumor promoting effects of glucagon receptor: a promising biomarker of papillary thyroid carcinoma via regulating EMT and P38/ERK pathways. *Hum. Cell* 33 175–184. 10.1007/s13577-019-00284-y 31782107

[B29] Kamps R. Brandão R. D. Bosch B. J. Paulussen A. D. C. Xanthoulea S. Blok M. J. (2017). Next-generation sequencing in oncology: genetic diagnosis, risk prediction and cancer classification. *Int. J. Mol. Sci.* 18:308. 10.3390/ijms18020308 28146134 PMC5343844

[B30] Katoh M. (2016). FGFR inhibitors: effects on cancer cells, tumor microenvironment and whole-body homeostasis (Review). *Int. J. Mol. Med.* 38 3–15. 10.3892/ijmm.2016.2620 27245147 PMC4899036

[B31] Kir S. White J. P. Kleiner S. Kazak L. Cohen P. Baracos V. E. (2014). Tumour-derived PTH-related protein triggers adipose tissue browning and cancer cachexia. *Nature* 513 100–104. 10.1038/nature13528 25043053 PMC4224962

[B32] Kobold S. Pantelyushin S. Rataj F. Vom Berg J. (2018). Rationale for combining bispecific T Cell activating antibodies with checkpoint blockade for cancer therapy. *Front. Oncol.* 8:285. 10.3389/fonc.2018.00285 30090763 PMC6068270

[B33] Kotteas E. A. Boulas P. Gkiozos I. Tsagkouli S. Tsoukalas G. Syrigos K. N. (2014). The intercellular cell adhesion molecule-1 (icam-1) in lung cancer: implications for disease progression and prognosis. *Anticancer Res.* 34 4665–4672. 25202042

[B34] Lang L. Teng Y. (2019). Fibroblast growth factor receptor 4 targeting in cancer: new insights into mechanisms and therapeutic strategies. *Cells* 8:31. 10.3390/cells8010031 30634399 PMC6356571

[B35] Langenfeld E. M. Bojnowski J. Perone J. Langenfeld J. (2005). Expression of bone morphogenetic proteins in human lung carcinomas. *Ann. Thorac. Surg.* 80 1028–1032. 10.1016/j.athoracsur.2005.03.094 16122479

[B36] Li B. Cui Y. Diehn M. Li R. (2017a). Development and validation of an individualized immune prognostic signature in early-stage nonsquamous non-small cell lung cancer. *JAMA Oncol.* 3 1529–1537. 28687838 10.1001/jamaoncol.2017.1609 PMC5710196

[B37] Li J. Wang J. Chen Y. Yang L. Chen S. (2017b). A prognostic 4-gene expression signature for squamous cell lung carcinoma. *J. Cell Physiol.* 232 3702–3713. 10.1002/jcp.25846 28160492

[B38] Lin P. Guo Y. N. Shi L. Li X. J. Yang H. He Y. (2019). Development of a prognostic index based on an immunogenomic landscape analysis of papillary thyroid cancer. *Aging.* 11 480–500. 10.18632/aging.101754 30661062 PMC6366981

[B39] Lin W. W. Karin M. (2007). A cytokine-mediated link between innate immunity, inflammation, and cancer. *J. Clin. Invest.* 117 1175–1183. 10.1172/jci31537 17476347 PMC1857251

[B40] Lin Y. C. Shun C. T. Wu M. S. Chen C. C. (2006). A novel anticancer effect of thalidomide: inhibition of intercellular adhesion molecule-1-mediated cell invasion and metastasis through suppression of nuclear factor-kappaB. *Clin. Cancer Res.* 12 7165–7173. 10.1158/1078-0432.ccr-06-1393 17145842

[B41] Lippitz B. E. (2013). Cytokine patterns in patients with cancer: a systematic review. *Lancet Oncol.* 14 e218–e228. 10.1016/s1470-2045(12)70582-x 23639322

[B42] Liu D. Schilling B. Liu D. Sucker A. Livingstone E. Jerby-Amon L. (2019). Integrative molecular and clinical modeling of clinical outcomes to PD1 blockade in patients with metastatic melanoma. *Nat. Med.* 25 1916–1927. 10.1038/s41591-019-0654-5 31792460 PMC6898788

[B43] Liu Y. Zhi Y. Song H. Zong M. Yi J. Mao G. (2019). S1PR1 promotes proliferation and inhibits apoptosis of esophageal squamous cell carcinoma through activating STAT3 pathway. *J. Exp. Clin. Cancer Res.* 38:369. 10.1186/s13046-019-1369-7 PMC6706905 31438989

[B44] Long J. Wang A. Bai Y. Lin J. Yang X. Wang D. (2019). Development and validation of a TP53-associated immune prognostic model for hepatocellular carcinoma. *EBioMedicine* 42 363–374. 10.1016/j.ebiom.2019.03.022 30885723 PMC6491941

[B45] Lu T. Yang X. Huang Y. Zhao M. Li M. Ma K. (2019). Trends in the incidence, treatment, and survival of patients with lung cancer in the last four decades. *Cancer Manag. Res.* 11 943–953. 10.2147/cmar.s187317 30718965 PMC6345192

[B46] Lu X. Yarbrough W. G. (2015). Negative regulation of RelA phosphorylation: emerging players and their roles in cancer. *Cytokine Growth Factor Rev.* 26 7–13. 10.1016/j.cytogfr.2014.09.003 25438737

[B47] Mackay L. K. Braun A. Macleod B. L. Collins N. Tebartz C. Bedoui S. (2015). Cutting edge: CD69 interference with sphingosine-1-phosphate receptor function regulates peripheral T cell retention. *J. Immunol.* 194 2059–2063. 10.4049/jimmunol.1402256 25624457

[B48] Mei S. Meyer C. A. Zheng R. Qin Q. Wu Q. Jiang P. (2017). Cistrome cancer: a web resource for integrative gene regulation modeling in cancer. *Cancer Res.* 77 e19–e22. 10.1158/0008-5472.can-17-0327 29092931 PMC5826647

[B49] Mohanty R. Chowdhury C. R. Arega S. Sen P. Ganguly P. Ganguly N. (2019). CAR T cell therapy: A new era for cancer treatment (Review). *Oncol. Rep.* 42 2183–2195. 31578576 10.3892/or.2019.7335

[B50] Newman A. M. Liu C. L. Green M. R. Gentles A. J. Feng W. Xu Y. (2015). Robust enumeration of cell subsets from tissue expression profiles. *Nat. Methods* 12 453–457. 10.1038/nmeth.3337 25822800 PMC4739640

[B51] No Authors Listed (2016). First anti-PD-L1 drug approved for NSCLC. *Cancer Discov.* 6:OF1. 10.1158/2159-8290.CD-NB2016-143 27920140

[B52] Ozawa Y. Amano Y. Kanata K. Hasegwa H. Matsui T. Kakutani T. (2019). Impact of early inflammatory cytokine elevation after commencement of PD-1 inhibitors to predict efficacy in patients with non-small cell lung cancer. *Med. Oncol.* 36:33. 10.1007/s12032-019-1255-3 30825015

[B53] Pinto M. P. Balmaceda C. Bravo M. L. Kato S. Villarroel A. Owen G. I. (2018). Patient inflammatory status and CD4+/CD8+ intraepithelial tumor lymphocyte infiltration are predictors of outcomes in high-grade serous ovarian cancer. *Gynecol. Oncol.* 151 10–17. 10.1016/j.ygyno.2018.07.025 30078505

[B54] Pons-Tostivint E. Thibault B. Guillermet-Guibert J. (2017). Targeting PI3K signaling in combination cancer therapy. *Trends Cancer* 3 454–469. 10.1016/j.trecan.2017.04.002 28718419

[B55] Popovic A. Jaffee E. M. Zaidi N. (2018). Emerging strategies for combination checkpoint modulators in cancer immunotherapy. *J. Clin. Invest.* 128 3209–3218. 10.1172/jci120775 30067248 PMC6063475

[B56] Qi L. Song W. Li L. Cao L. Yu Y. Song C. (2016). FGF_4_ induces epithelial-mesenchymal transition by inducing store-operated calcium entry in lung adenocarcinoma. *Oncotarget* 7 74015–74030. 10.18632/oncotarget.12187 27677589 PMC5342032

[B57] Rajski M. Saaf A. Buess M. (2015). BMP2 response pattern in human lung fibroblasts predicts outcome in lung adenocarcinomas. *BMC Med. Genomics* 8:16. 10.1186/s12920-015-0090-4 25924783 PMC4422073

[B58] Rivas-Fuentes S. Salgado-Aguayo A. Pertuz Belloso S. Gorocica Rosete P. Alvarado-Vasquez N. Aquino-Jarquin G. (2015). Role of chemokines in non-small cell lung cancer: angiogenesis and inflammation. *J. Cancer* 6 938–952. 10.7150/jca.12286 26316890 PMC4543754

[B59] Robinson M. D. McCarthy D. J. Smyth G. K. (2010). edgeR: a bioconductor package for differential expression analysis of digital gene expression data. *Bioinformatics* 26 139–140. 10.1093/bioinformatics/btp616 19910308 PMC2796818

[B60] Shannon P. Markiel A. Ozier O. Baliga N. S. Wang J. T. Ramage D. (2003). Cytoscape: a software environment for integrated models of biomolecular interaction networks. *Genome Res.* 13 2498–2504. 10.1101/gr.1239303 14597658 PMC403769

[B61] Siegel R. L. Miller K. D. Jemal A. (2020). Cancer statistics. *CA Cancer J. Clin.* 70 7–30. 10.3322/caac.21590 31912902

[B62] Siow Z. R. De Boer R. H. Lindeman G. J. Mann G. B. (2018). Spotlight on the utility of the Oncotype DX((R)) breast cancer assay. *Int. J. Womens Health* 10 89–100. 10.2147/ijwh.s124520 29503586 PMC5827461

[B63] Song B. N. Kim S. K. Mun J. Y. Choi Y. D. Leem S. H. Chu I. S. (2019). Identification of an immunotherapy-responsive molecular subtype of bladder cancer. *EBioMedicine* 50 238–245. 10.1016/j.ebiom.2019.10.058 31735557 PMC6921227

[B64] Sul J. Blumenthal G. M. Jiang X. He K. Keegan P. Pazdur R. (2016). FDA approval summary: pembrolizumab for the treatment of patients with metastatic non-small cell lung cancer whose tumors express programmed death-ligand 1. *Oncologist* 21 643–650. 10.1634/theoncologist.2015-0498 27026676 PMC4861368

[B65] Tan I. B. Tan P. (2011). Genetics: an 18-gene signature (ColoPrint(R)) for colon cancer prognosis. *Nat. Rev. Clin. Oncol.* 8 131–133. 10.1038/nrclinonc.2010.229 21304502

[B66] Tan L. M. Li X. Qiu C. F. Zhu T. Hu C. P. Yin J. Y. (2019). CLEC4M is associated with poor prognosis and promotes cisplatin resistance in NSCLC patients. *J. Cancer* 10 6374–6383. 10.7150/jca.30139 31772670 PMC6856750

[B67] Tanaka T. Sho M. Takayama T. Wakatsuki K. Matsumoto S. Migita K. (2014). Endothelin B receptor expression correlates with tumour angiogenesis and prognosis in oesophageal squamous cell carcinoma. *Br. J. Cancer* 110 1027–1033. 10.1038/bjc.2013.784 24357795 PMC3929870

[B68] Thomas A. Chen Y. Yu T. Jakopovic M. Giaccone G. (2015). Trends and characteristics of young non-small cell lung cancer patients in the United States. *Front. Oncol.* 5:113. 10.3389/fonc.2015.00113 26075181 PMC4443720

[B69] Travis W. D. (2020). Lung cancer pathology: current concepts. *Clin. Chest Med.* 41 67–85. 32008630 10.1016/j.ccm.2019.11.001

[B70] Vrieze S. I. (2012). Model selection and psychological theory: a discussion of the differences between the Akaike information criterion (AIC) and the Bayesian information criterion (BIC). *Psychol. Methods* 17 228–243. 10.1037/a0027127 22309957 PMC3366160

[B71] Wan Y. W. Raese R. A. Fortney J. E. Xiao C. Luo D. Cavendish J. (2012). A smoking-associated 7-gene signature for lung cancer diagnosis and prognosis. *Int. J. Oncol.* 41 1387–1396. 22825454 10.3892/ijo.2012.1556 PMC3481011

[B72] Wang C. Chen Z. Bu X. Han Y. Shan S. Ren T. (2016). IL-33 signaling fuels outgrowth and metastasis of human lung cancer. *Biochem. Biophys. Res. Commun.* 479 461–468. 10.1016/j.bbrc.2016.09.081 27644880

[B73] Wang Y. Zhang X. Yang L. Xue J. Hu G. (2018). Blockade of CCL2 enhances immunotherapeutic effect of anti-PD1 in lung cancer. *J. Bone Oncol.* 11 27–32. 10.1016/j.jbo.2018.01.002 29892522 PMC5993943

[B74] Wang Z. Song Q. Yang Z. Chen J. Shang J. Ju W. (2019). Construction of immune-related risk signature for renal papillary cell carcinoma. *Cancer Med.* 8 289–304. 10.1002/cam4.1905 30516029 PMC6346237

[B75] Wei S. C. Duffy C. R. Allison J. P. (2018). Fundamental mechanisms of immune checkpoint blockade therapy. *Cancer Discov.* 8 1069–1086. 10.1158/2159-8290.cd-18-0367 30115704

[B76] Woo S. R. Turnis M. E. Goldberg M. V. Bankoti J. Selby M. Nirschl C. J. (2012). Immune inhibitory molecules LAG-3 and PD-1 synergistically regulate T-cell function to promote tumoral immune escape. *Cancer Res.* 72 917–927. 10.1158/0008-5472.can-11-1620 22186141 PMC3288154

[B77] Xiao L. Lan X. Shi X. Zhao K. Wang D. Wang X. (2017). Cytoplasmic RAP1 mediates cisplatin resistance of non-small cell lung cancer. *Cell Death Dis.* 8:e2803. 10.1038/cddis.2017.210 28518145 PMC5520727

[B78] Yan Z. Wang Q. Lu Z. Sun X. Song P. Dang Y. (2020). OSluca: an interactive web server to evaluate prognostic biomarkers for lung cancer. *Front. Genet.* 11:420. 10.3389/fgene.2020.00420 32528519 PMC7264384

[B79] Yang X. Peng P. Zhang L. (2019). Multiline treatment of advanced squamous cell carcinoma of the lung: a case report and review of the literature. *World J. Clin. Cases* 7 1899–1907. 10.12998/wjcc.v7.i14.1899 31417937 PMC6692274

[B80] Yang Z. Gao X. Wang J. Xu L. Zheng Y. Xu Y. (2018). Interleukin-33 enhanced the migration and invasiveness of human lung cancer cells. *Onco Targets Ther.* 11 843–849. 10.2147/ott.s155905 29497316 PMC5820469

[B81] Yu G. Wang L. G. Han Y. He Q. Y. (2012). clusterProfiler: an R package for comparing biological themes among gene clusters. *OMICS* 16 284–287. 10.1089/omi.2011.0118 22455463 PMC3339379

[B82] Zhang L. Wang J. Wei F. Wang K. Sun Q. Yang F. (2016). Profiling the dynamic expression of checkpoint molecules on cytokine-induced killer cells from non-small-cell lung cancer patients. *Oncotarget* 7 43604–43615. 10.18632/oncotarget.9871 27283895 PMC5190047

[B83] Zhang X. Zeng Y. Qu Q. Zhu J. Liu Z. Ning W. (2017). PD-L1 induced by IFN-gamma from tumor-associated macrophages via the JAK/STAT3 and PI3K/AKT signaling pathways promoted progression of lung cancer. *Int. J. Clin. Oncol.* 22 1026–1033. 10.1007/s10147-017-1161-7 28748356

[B84] Zhang Y. Roth J. A. Yu H. Ye Y. Xie K. Zhao H. (2019). A 5-microRNA signature identified from serum microRNA profiling predicts survival in patients with advanced stage non-small cell lung cancer. *Carcinogenesis* 40 643–650. 10.1093/carcin/bgy132 30428030 PMC6610172

[B85] Zhao Y. Ma K. Yang S. Zhang X. Wang F. Zhang X. (2018). MicroRNA-125a-5p enhances the sensitivity of esophageal squamous cell carcinoma cells to cisplatin by suppressing the activation of the STAT3 signaling pathway. *Int. J. Oncol.* 53 644–658. 10.3892/ijo.2018.4409 29767234 PMC6017156

[B86] Zhou J. Qu Z. Yan S. Sun F. Whitsett J. A. Shapiro S. D. (2015). Differential roles of STAT3 in the initiation and growth of lung cancer. *Oncogene* 34 3804–3814. 10.1038/onc.2014.318 25284582 PMC4387125

