# Supplementary material for: Development of an Immune-Related Risk Signature for Predicting Prognosis in Lung Squamous Cell Carcinoma
Source: Front Genet. 2020 Aug 28;11:978. doi: 10.3389/fgene.2020.00978 (PMC7485220; doi:10.3389/fgene.2020.00978)
Supplement: Supplementary file 2 [file Table_2.doc]

**Table S2. The risk scores and clinical pathological characteristics for LSCC patients from TCGA, and GSE4573 and GSE17710.**

| **Study ID** | **Training cohorts** | | **Validation cohorts** | | | |
| --- | --- | --- | --- | --- | --- | --- |
|  |  |  |  |  |  |  |
|  |  |  |  |  |  |  |
|  | TCGA samples | Risk score (P < 0.05) | GSE4573 | Risk score (P < 0.05) | GSE17710 | Risk score (P < 0.05) |
| **Platform** | Illumina HiSeq RNA Seq |  | Affymetrix Human Genome U133A Array |  | Agilent-UNC-custom-4X44K |  |
| **Total N** | 475 |  | 130 |  | 56 |  |
| **Age(years)** | | | | | |  |
| ≤60 | 113 | 0.0218 | 33 | 0.2790 | 13 | 0.1317 |
| >60 | 362 |  | 97 |  | 43 |  |
| **pT** | | | | | |  |
| T1 | 108 | 0.9912^a^ | 33 | 0.3022 | NA | NA |
| T2 | 278 |  | 76 |  |  |  |
| T3 | 68 |  | 15 |  |  |  |
| T4 | 21 |  | 6 |  |  |  |
| **pN** | | | | | |  |
| N0 | 301 | 0.3046^b^ | 83 | 0.7094 | NA | NA |
| N1 | 125 |  | 32 |  |  |  |
| N2 | 39 |  | 15 |  |  |  |
| N3 | 5 |  | 0 |  |  |  |
| Nx | 5 |  | 0 |  |  |  |
| **pM** | | | | | |  |
| M0 | 395 | 0.6697^c^ | 129 | <0.0001 | NA | NA |
| M1 | 6 |  | 0 |  |  |  |
| M2 | 0 |  | 0 |  |  |  |
| M3 | 0 |  | 0 |  |  |  |
| M4 | 0 |  | 0 |  |  |  |
| Mx | 73 |  | 0 |  |  |  |
| NA | 1 |  | 1 |  |  |  |
| **Clinical Stage** | | | | | |  |
| I | 228 | 0.9666^d^ | 73 | 0.0438 | 34 | 0.7408 |
| II | 156 |  | 34 |  | 19 |  |
| III | 81 |  | 23 |  | 3 |  |
| IV | 6 |  | 0 |  | 0 |  |
| NA | 4 |  | 0 |  | 0 |  |
| **Differentiation** | | | | | |  |
| Well | NA | NA | 1 | 0.3416^e^ | 0 | 0.6304 |
| Well-moderate |  |  | 14 |  | 0 |  |
| Moderate |  |  | 59 |  | 36 |  |
| Moderate-poor |  |  | 34 |  | 0 |  |
| Poor |  |  | 22 |  | 20 |  |
| **Sex** | | | | | |  |
| Female | 123 | 0.1531 | 48 | 0.7083 | 24 | 0.9386 |
| Male | 352 |  | 82 |  | 32 |  |
| **Survival Status** | | | | | |  |
| Alive | 279 | <0.0001 | 63 | 0.8866 | 22 | 0.6410 |
| Dead | 196 |  | 67 |  | 34 |  |

**Note:** a, Two-tailed T test of pT1-pT2 and pT3-pT4; b, Two-tailed T test of pN0 and pN1-pNx; c, Two-tailed T test of pM0 and pM1-pMx; d, Two-tailed T test of clinical Stage T1-T2 and T3-T4; e, Two-tailed T test of Well and Moderate differentiation and poor differentiation.
