# Supplementary material for: Development of an Immune-Related Risk Signature for Predicting Prognosis in Lung Squamous Cell Carcinoma
Source: Front Genet. 2020 Aug 28;11:978. doi: 10.3389/fgene.2020.00978 (PMC7485220; doi:10.3389/fgene.2020.00978)
Supplement: Supplementary file 3 [file Table_3.docx]

**Table S3. 50 differentially expressed TFs and their prognostic utility in LSCC patients (*P* < 0.05).**

| **Gene** | **logFC** | ***P*-Value** | **FDR** | **HR** | **Z** | ***P*-value** |
| --- | --- | --- | --- | --- | --- | --- |
| TCF21 | -4.59677 | 1.13E-213 | 1.04E-210 | 1.120168 | 2.48093 | 0.013104 |
| HNF1B | -3.61162 | 3.62E-57 | 9.23E-56 | 1.068671 | 2.242323 | 0.02494 |
| SOX2 | 5.191735 | 2.05E-42 | 3.24E-41 | 0.947631 | -2.13713 | 0.032588 |
| FOXA2 | -3.30629 | 1.56E-50 | 3.29E-49 | 1.062046 | 1.924763 | 0.054259 |
| GATA6 | -2.63009 | 8.58E-92 | 5.16E-90 | 1.12162 | 1.854813 | 0.063623 |
| CDX2 | 6.086212 | 1.21E-16 | 5.50E-16 | 1.045563 | 1.818839 | 0.068936 |
| TP63 | 5.731034 | 1.39E-64 | 4.42E-63 | 0.952075 | -1.81654 | 0.069287 |
| SOX17 | -3.4982 | 5.68E-189 | 2.62E-186 | 1.12508 | 1.783295 | 0.074538 |
| NFE2 | -2.18293 | 1.70E-29 | 1.55E-28 | 1.074435 | 1.684257 | 0.092132 |
| LHX2 | 6.481348 | 7.41E-41 | 1.11E-39 | 0.957473 | -1.40873 | 0.158915 |
| GATA2 | -2.13484 | 2.69E-46 | 4.88E-45 | 1.080562 | 1.406662 | 0.159528 |
| EZH2 | 3.17019 | 1.28E-91 | 7.64E-90 | 0.893151 | -1.39398 | 0.163324 |
| EMX1 | 5.827603 | 1.73E-33 | 1.89E-32 | 0.960896 | -1.36445 | 0.172426 |
| SCML2 | 2.193419 | 1.51E-17 | 7.27E-17 | 0.945585 | -1.29576 | 0.19506 |
| NR5A2 | -2.38969 | 6.16E-92 | 3.73E-90 | 1.100326 | 1.286218 | 0.198367 |
| PAX3 | 6.036285 | 1.11E-21 | 6.78E-21 | 0.965203 | -1.17348 | 0.240604 |
| NR4A1 | -2.94431 | 9.09E-88 | 5.10E-86 | 1.06573 | 1.155145 | 0.248031 |
| TFAP2A | 5.360118 | 1.72E-121 | 2.02E-119 | 1.074278 | 1.148203 | 0.250885 |
| EPAS1 | -3.32706 | 2.19E-200 | 1.36E-197 | 1.078855 | 1.133414 | 0.257041 |
| HOXB13 | 8.257429 | 1.49E-27 | 1.23E-26 | 1.023754 | 1.126512 | 0.259949 |
| LMNB1 | 2.053834 | 2.14E-61 | 6.19E-60 | 0.897142 | -1.11346 | 0.265509 |
| HNF4G | 2.131118 | 3.44E-16 | 1.51E-15 | 1.044777 | 1.067303 | 0.285835 |
| CENPA | 4.655385 | 1.07E-157 | 2.80E-155 | 0.918293 | -1.0308 | 0.302635 |
| BCL11A | 3.182667 | 1.00E-40 | 1.49E-39 | 0.951356 | -1.00182 | 0.316429 |
| GATA4 | 2.694521 | 4.19E-07 | 9.30E-07 | 1.019611 | 0.85668 | 0.391622 |
| EPO | 2.890436 | 2.11E-15 | 8.82E-15 | 0.962196 | -0.8319 | 0.405464 |
| HOXC11 | 7.989583 | 6.14E-40 | 8.85E-39 | 1.020444 | 0.821143 | 0.411565 |
| HOXC9 | 3.65707 | 4.30E-28 | 3.65E-27 | 1.027792 | 0.792322 | 0.428173 |
| EGR1 | -2.04142 | 2.69E-44 | 4.54E-43 | 1.045692 | 0.769474 | 0.441612 |
| FLI1 | -2.30625 | 2.73E-85 | 1.42E-83 | 1.052261 | 0.75363 | 0.451071 |
| FOS | -2.45662 | 7.72E-55 | 1.83E-53 | 1.039737 | 0.749306 | 0.453673 |
| H2AFX | 2.00995 | 8.97E-54 | 2.07E-52 | 0.94073 | -0.67871 | 0.49732 |
| PDX1 | 6.15166 | 3.42E-18 | 1.71E-17 | 1.019377 | 0.646166 | 0.518172 |
| SALL4 | 3.967049 | 6.65E-42 | 1.04E-40 | 1.029608 | 0.617294 | 0.537041 |
| RBP2 | -3.78772 | 2.98E-100 | 2.12E-98 | 1.03039 | 0.612522 | 0.540192 |
| HOXA9 | 2.211823 | 5.74E-13 | 2.04E-12 | 1.023942 | 0.567183 | 0.57059 |
| TFAP2C | 2.303508 | 1.10E-44 | 1.89E-43 | 0.96935 | -0.42528 | 0.67063 |
| FOXM1 | 4.442086 | 2.32E-115 | 2.34E-113 | 1.029786 | 0.384105 | 0.700901 |
| CBX2 | 3.381152 | 2.33E-43 | 3.82E-42 | 0.982792 | -0.35561 | 0.722135 |
| ASCL1 | 5.73429 | 8.91E-12 | 2.90E-11 | 1.009189 | 0.32435 | 0.745673 |
| RXRG | -3.22222 | 2.45E-55 | 5.91E-54 | 0.988077 | -0.29729 | 0.766246 |
| NR2F1 | -2.09749 | 1.40E-66 | 4.64E-65 | 1.018491 | 0.289945 | 0.771858 |
| TAL1 | -3.0944 | 2.56E-104 | 2.02E-102 | 1.015386 | 0.281286 | 0.778491 |
| HOXB7 | 2.586554 | 7.20E-33 | 7.65E-32 | 1.013355 | 0.271787 | 0.785786 |
| ERG | -2.55055 | 1.38E-137 | 2.32E-135 | 1.016973 | 0.201877 | 0.840013 |
| MYBL2 | 4.741501 | 1.18E-136 | 1.95E-134 | 1.013803 | 0.166959 | 0.867402 |
| TP73 | 2.139196 | 5.30E-19 | 2.78E-18 | 1.00595 | 0.124047 | 0.901278 |
| NCAPG | 3.656397 | 1.35E-127 | 1.81E-125 | 0.992153 | -0.07801 | 0.937821 |
| E2F7 | 4.531177 | 1.56E-103 | 1.20E-101 | 0.995722 | -0.06674 | 0.94679 |
| RUNX1T1 | -2.05561 | 7.51E-42 | 1.17E-40 | 0.997091 | -0.05658 | 0.95488 |
