# Supplementary material for: Development of an Immune-Related Risk Signature for Predicting Prognosis in Lung Squamous Cell Carcinoma
Source: Front Genet. 2020 Aug 28;11:978. doi: 10.3389/fgene.2020.00978 (PMC7485220; doi:10.3389/fgene.2020.00978)
Supplement: Supplementary file 4 [file Data_Sheet_1.docx]

Supplementary Material

**Supplementary Figures**

**Figure S1. Identification of all differentially expressed genes between normal and tumor cases.**

(A). The significant enriched biological processes. (B). The significant enriched molecular functions. (C). The significant enriched cellular components. (D). The significant enriched KEGG pathways.

**Figure S2. Model gene expression in high- and low-risk groups.**

(A). GCGR; (B). FGF8; (C). CLEC4M; (D).PTH; (E). SLC10A2; (F). NPPC; (G). FGF4.

**Figure S3. Model gene expression in normal and LSCC cases.**

(A). GCGR; (B). FGF8; (C). CLEC4M; (D).PTH; (E). SLC10A2; (F). NPPC; (G). FGF4.

**Figure S4. Tumor infiltration levels of the model genes among the somatic copy number alternations category in 6 immune cell subsets.**

(A). GCGR; (B). FGF8; (C). CLEC4M; (D).PTH; (E). SLC10A2; (F). NPPC; (G). FGF4.

**Figure S5. The difference of risk score in high- and low-risk patients groups with different clinical parameters.**

(A). Clinical tumor stages; (B). Node status; (C). T stages; (D). M status.

**Figure S6. Tumor cell infiltration levels of the model genes in 6 immune cell subsets.**

(A). GCGR; (B). FGF8; (C). CLEC4M; (D).PTH; (E). SLC10A2; (F). NPPC; (G). FGF4.

**Figure S7. The prognostic significance of the seven model gene expression levels in LSCC patients (OSluca database).**

(A). GCGR; (B). FGF8; (C). CLEC4M; (D).PTH; (E). SLC10A2; (F). NPPC; (G). FGF4.

**Supplementary Tables**

**Table S1. Immune related genes from Immunology Database and Analysis Portal.**

**Table S2. The risk scores and clinical pathological characteristics for LSCC patients from TCGA, and GSE4573 and GSE17710.**

**Table S3. 50 differentially expressed TFs and their prognostic utility in LSCC patients (*P* < 0.05).**
