# Supplementary figures and images for: Development of an Immune-Related Risk Signature for Predicting Prognosis in Lung Squamous Cell Carcinoma
Source: Front Genet. 2020 Aug 28;11:978. doi: 10.3389/fgene.2020.00978 (PMC7485220; doi:10.3389/fgene.2020.00978)

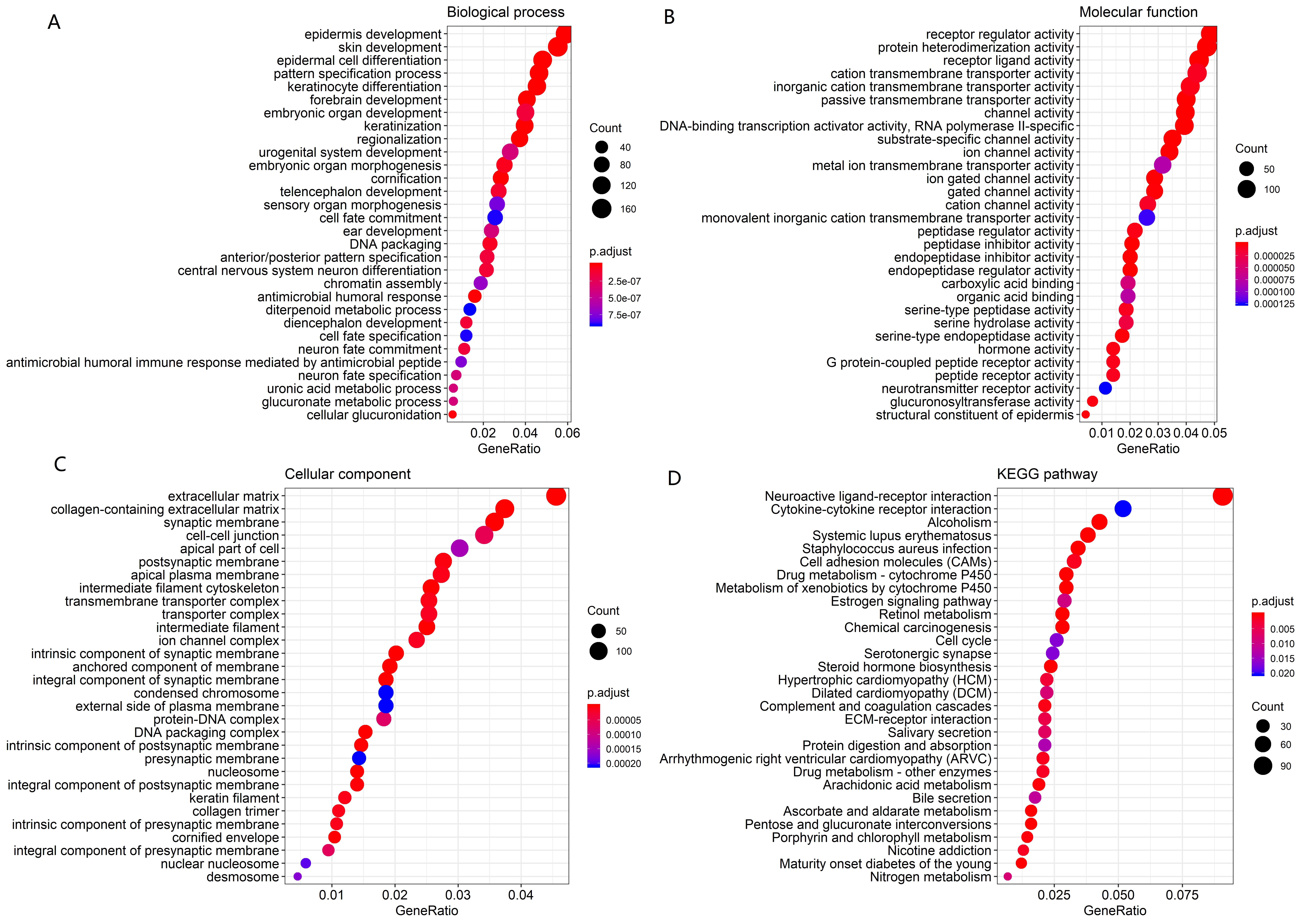

Supplement: Supplementary file 5 [file Image_1.TIFF]

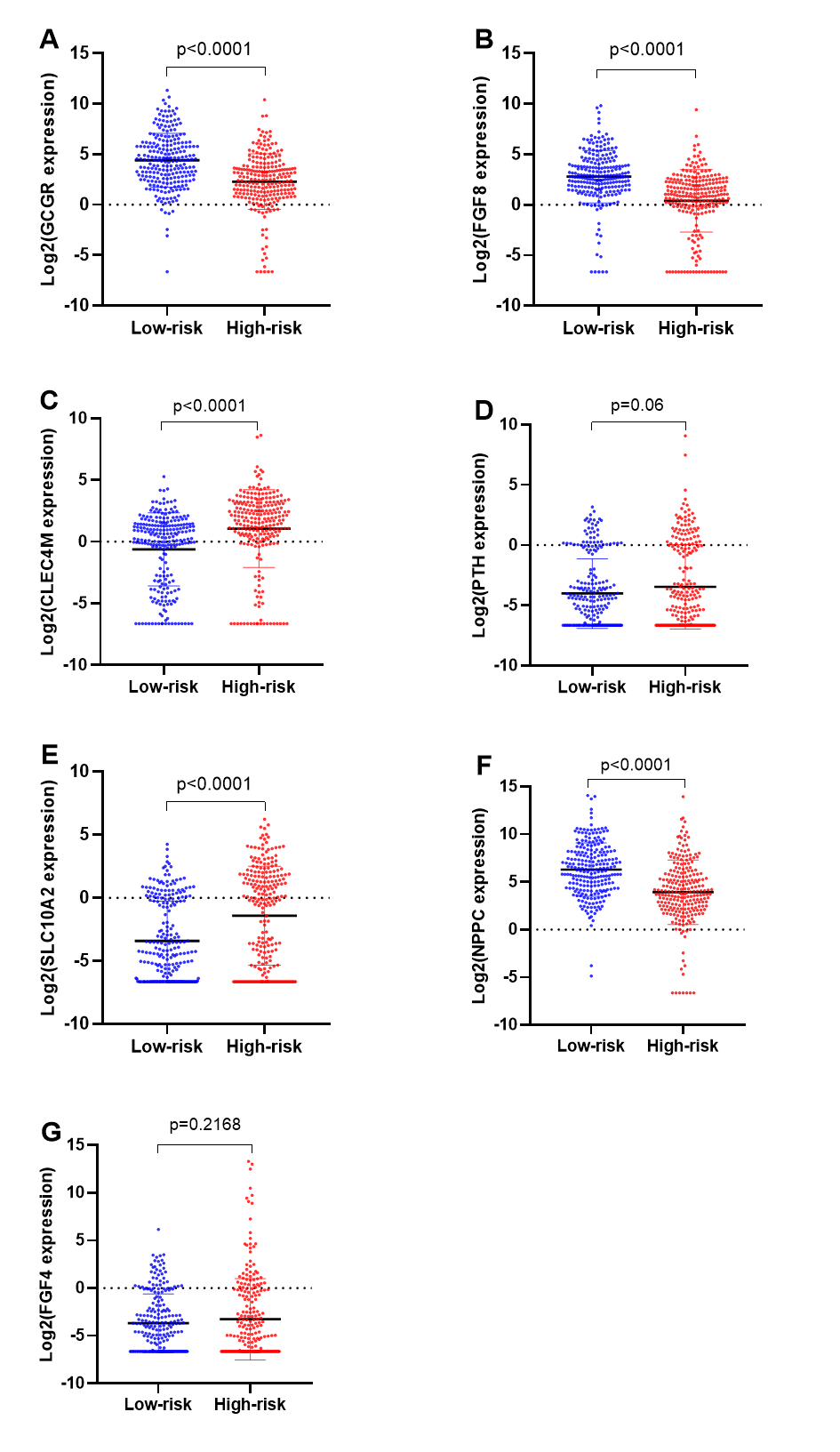

Supplement: Supplementary file 6 [file Image_2.TIF]

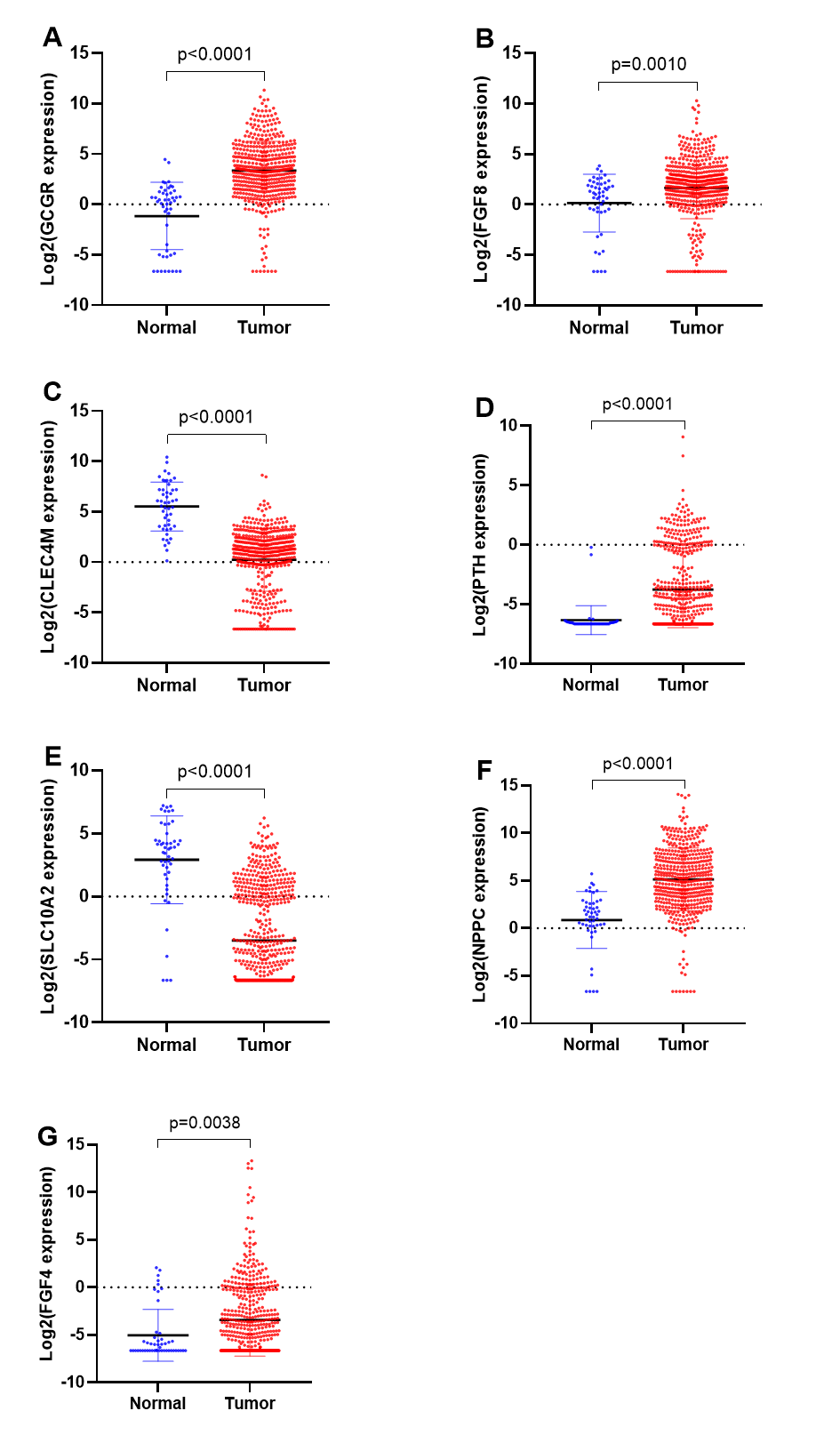

Supplement: Supplementary file 7 [file Image_3.TIF]

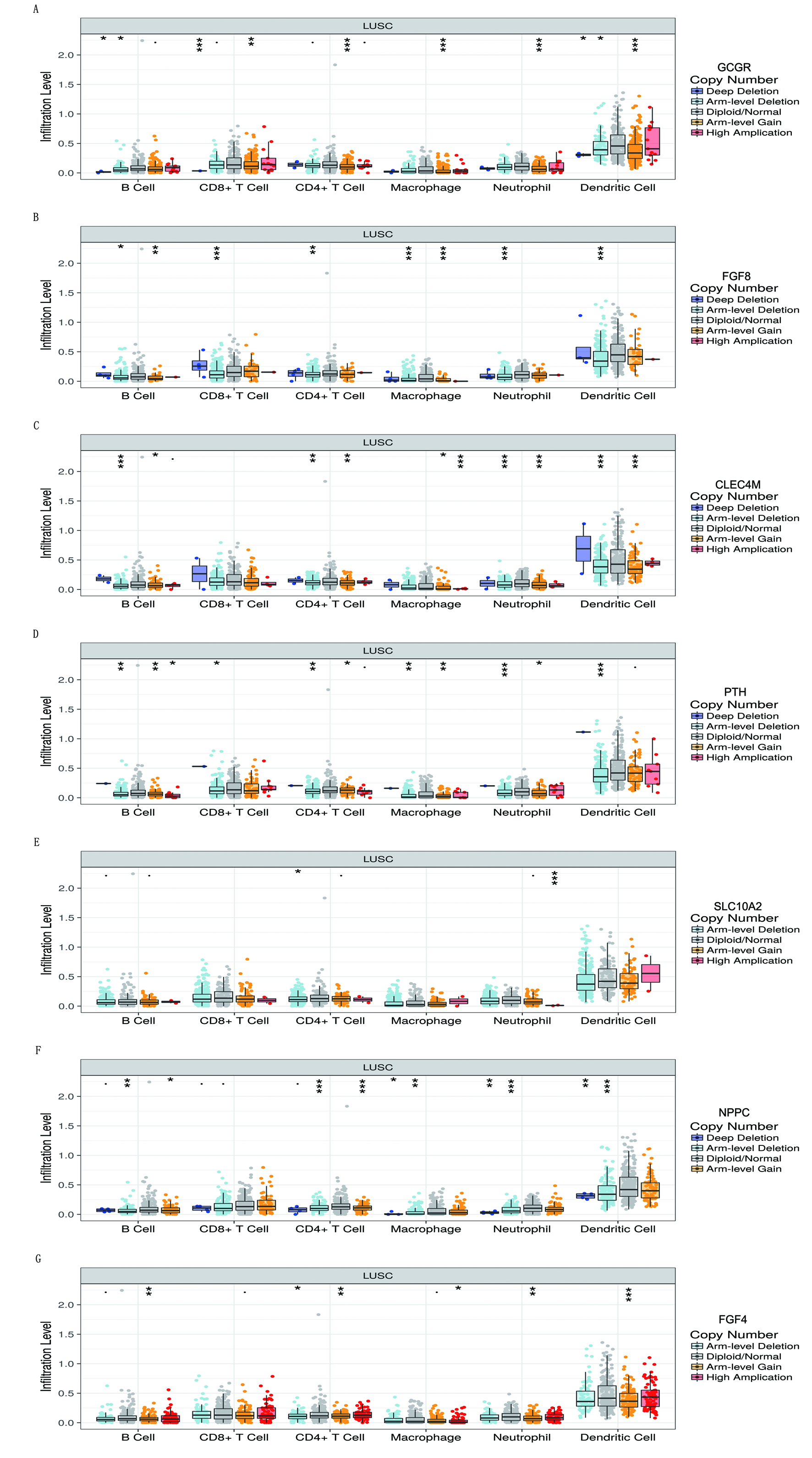

Supplement: Supplementary file 8 [file Image_4.TIF]

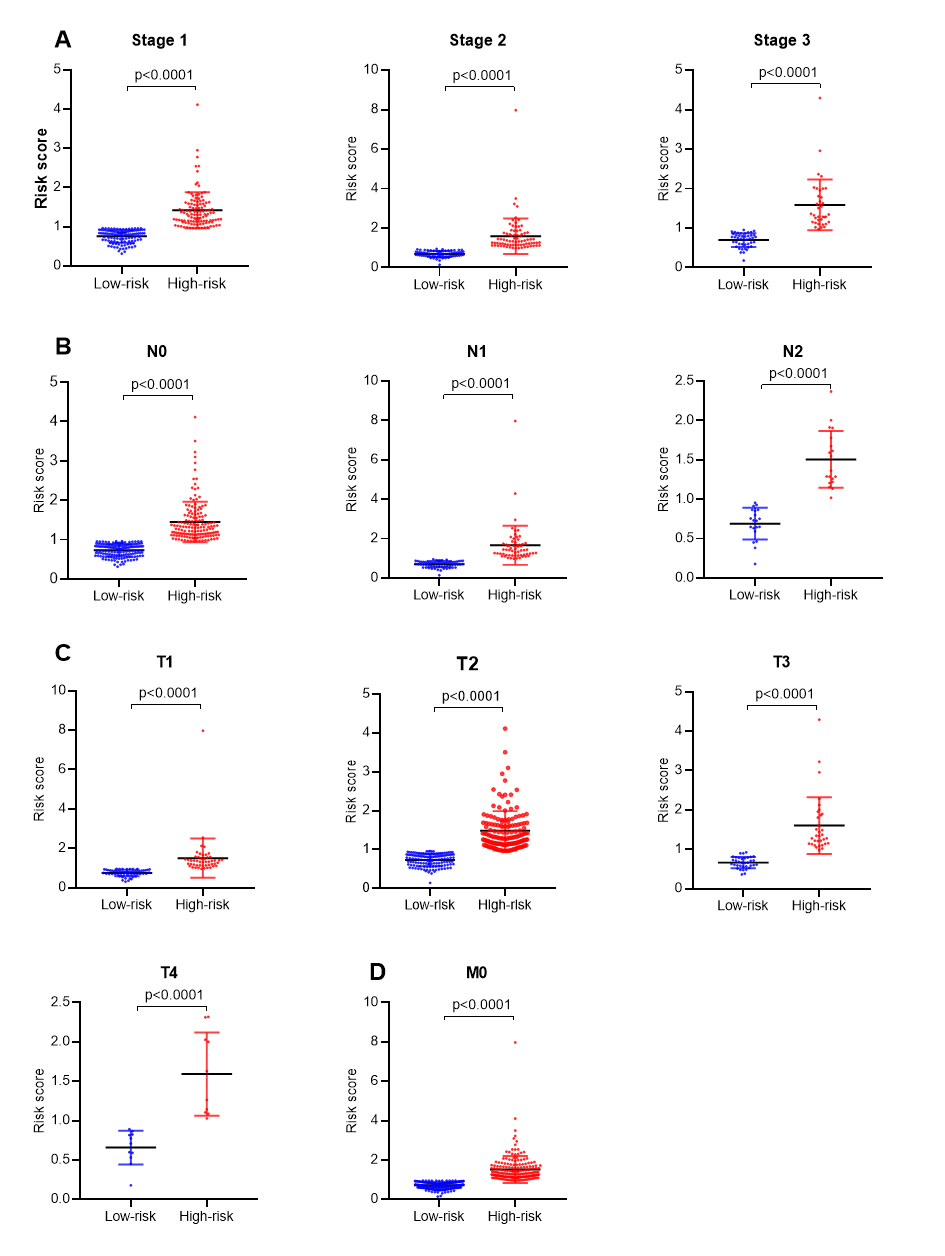

Supplement: Supplementary file 9 [file Image_5.TIF]

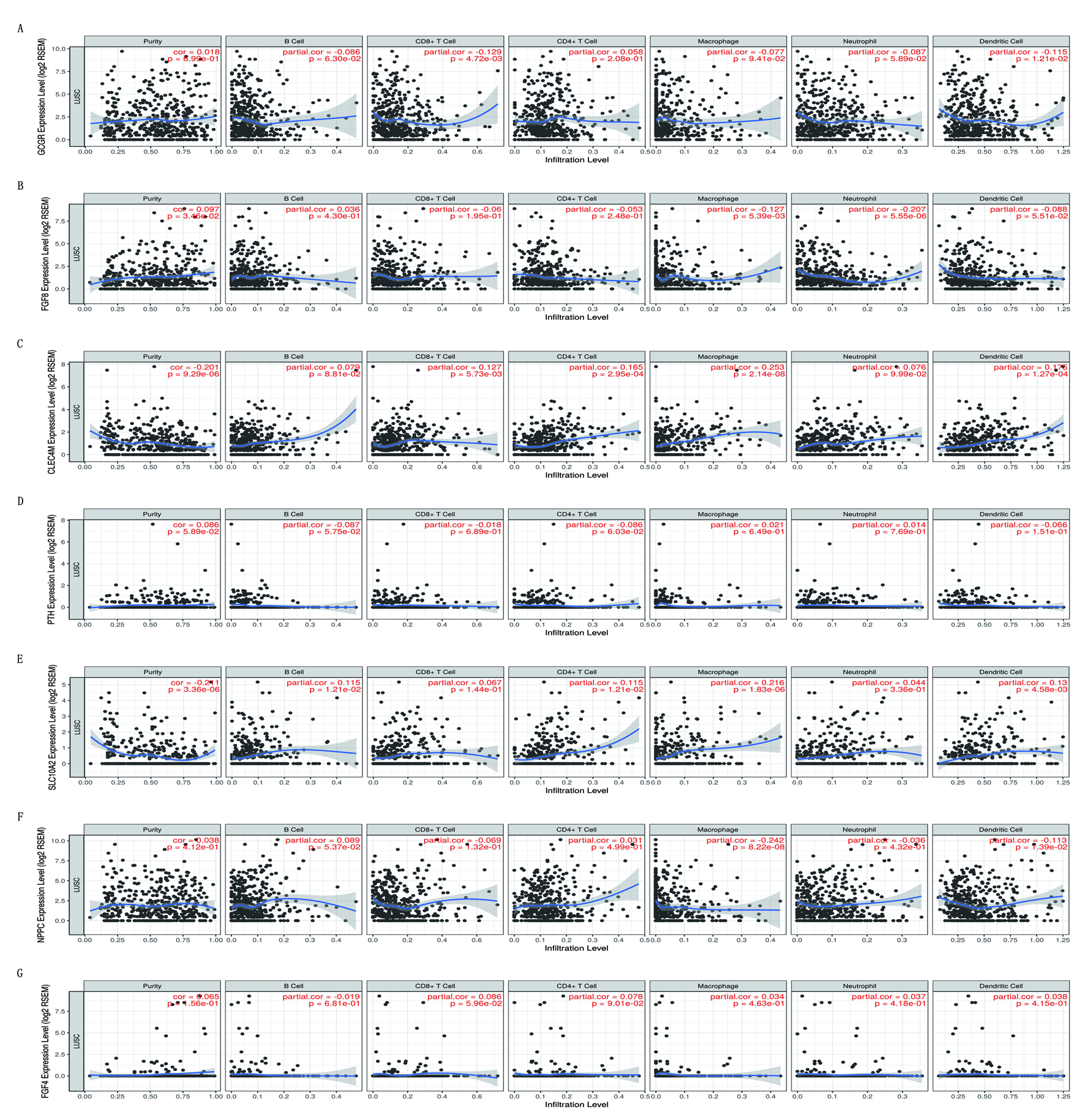

Supplement: Supplementary file 10 [file Image_6.TIF]

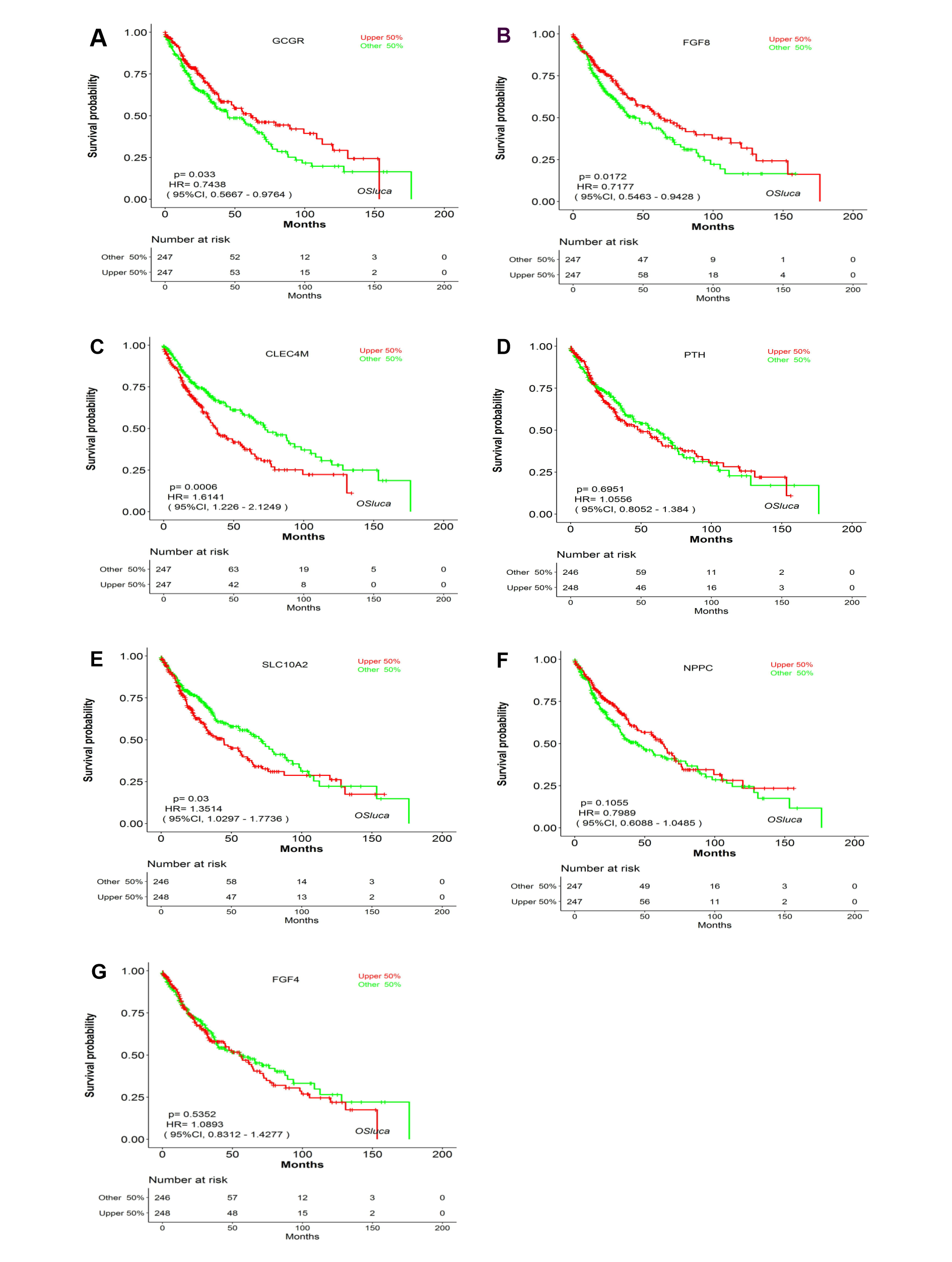

Supplement: Supplementary file 11 [file Image_7.TIF]
